# Unraveling the contributions of prosodic patterns and individual traits on cross-linguistic perception of Spanish sentence modality

**DOI:** 10.1371/journal.pone.0298708

**Published:** 2024-02-29

**Authors:** Peizhu Shang, Yuejiao Li, Yuhao Liang

**Affiliations:** 1 School of Foreign Languages, Beijing Institute of Technology, Beijing, China; 2 School of Foreign Studies, Hefei University of Technology, Anhui, China; 3 College of International Education, Guizhou University, Guiyang, China; 4 School of International Chinese Language Education, Beijing Normal University, Beijing, China; Cardiff University, UNITED KINGDOM

## Abstract

Cross-linguistic perception is known to be molded by native and second language (L2) experiences. Yet, the role of prosodic patterns and individual characteristics on how speakers of tonal languages perceive L2 Spanish sentence modalities remains relatively underexplored. This study addresses the gap by analyzing the auditory performance of 75 Mandarin speakers with varying levels of Spanish proficiency. The experiment consisted of four parts: the first three collected sociolinguistic profiles and assessed participants’ pragmatic competence and musical abilities. The last part involved an auditory gating task, where participants were asked to identify Spanish broad focus statements and information-seeking yes/no questions with different stress patterns. Results indicated that the shape of intonation contours and the position of the final stressed syllable significantly impact learners’ perceptual accuracy, with effects modulated by utterance length and L2 proficiency. Moreover, individual differences in pragmatic and musical competence were found to refine auditory and cognitive processing in Mandarin learners, thereby influencing their ability to discriminate question-statement contrasts. These findings reveal the complex interplay between prosodic and individual variations in L2 speech perception, providing novel insights into how speakers of tonal languages process intonation in a non-native Romance language like Spanish.

## 1 Introduction

The utilization of fundamental frequency (f0) differs between tonal and intonational languages, with tonal languages primarily using f0 modulation to convey lexical meanings, while intonational languages employ it to communicate postlexical information. For learners whose first language (L1) is tonal, such as Mandarin Chinese, acquiring an intonational language like Spanish as a second language (L2) often presents significant challenges due to these disparities [1–3]. Despite the extensive research on L2 perceptual acquisition, focusing on various speech components such as segments [4], stress [5, 6], prominence [7, 8], and lexical tones [9], limited attention has been given to the perception of sentence-type contrasts in contact situations involving tonal and non-tonal languages. Currently, most of the scientific literature examining L2 sentence perception by tonal language speakers has predominantly centered on Mandarin learners of English, mainly attributing cross-linguistic discrepancies to the influence of the learners’ L1 [10–13]. However, recent findings suggest that the ability of L2 listeners to process segmental and suprasegmental features of speech may be influenced by various interindividual factors, including biological characteristics [4, 14, 15], psychometric traits [7, 16], and music expertise [17, 18]. On the other hand, previous acoustic analyses of Spanish intonation have provided evidence of distinct intonation contours that categorically differentiate statements from questions, thereby offering prosodic cues that can enhance native listeners’ perceptual accuracy [19]. Additionally, other prosodic-related elements, such as the location of the final stressed syllable, have been theorized to impact the production of Spanish intonation by altering the trajectory of pitch movements [20]. Although the knowledge from previous works has contributed greatly to the current understanding of L2 speech perception, it remains unclear how these prosodic and individual factors interact and affect the auditory processing of L2 sentence modalities in Spanish for speakers of tonal languages.

The present study aims to address this research gap, based on the expanding importance of multilingualism in today’s globalized society, where individuals frequently learn an L2 that differs greatly from their L1 in phonetic and phonological dimensions. Specifically, this study focuses on examining how (a) prosodic elements, including intonation pattern and stress location, and (b) individual traits such as age, gender, pragmatic skills, and musical ability, impact the perception of Spanish sentence modalities by native speakers of Mandarin Chinese who have acquired Peninsular Spanish as an L2. By doing so, this study aims to unravel the complexities of L2 auditory processing at the sentence intonation level and potentially contribute to future design of more effective instructional materials and curricula for L2 Spanish learners from tonal languages. The remainder of the paper is structured as follows. The Introduction section reviews the relevant literature on L2 perception and outlines the research questions of the study. The Methods section describes the methodology employed in this study. The Results section presents the statistical results, followed by a discussion of the findings in the Discussion section. Finally, the paper concludes in the Conclusions section.

### 1.1 Prosodic cues influencing Spanish sentence perception

In Peninsular Spanish, distinguishing between statements and yes/no questions can be challenging due to similarities in word choice and grammar, prompting heavy reliance on intonation contours for discerning intended linguistic meanings [21–23]. Prior phonetic analyses have evidenced four intonation patterns categorically marking declarative versus interrogative modality in Spanish [19, 24]. The most salient is pitch movement at the utterance end, with final rises signaling questions and final falls indicating statements. This pattern can be recognized cross-linguistically by both native Spanish speakers and non-native speakers from various L1 backgrounds. For example, learners of Spanish with both Chinese and English as their L1 were found to be able to distinguish statements from yes/no questions based solely on the final rising or falling intonation contour [25, 26], suggesting potential prosodic universality for conveying sentence type. This may be determined by biological codes, e.g., the Frequency Code, which associates higher f0 with uncertainty, thereby serving as an interrogative marker, and lower f0 with assertiveness, marking declarative modality [27, 28].

While the final pitch contour strongly conveys the intonation category, early pitch movements in the prenuclear position also contribute to sentence type identification. Prior research found that the broad focus statement in Spanish exhibits an initial f0 rise on stressed syllables, with the first f0 peak occurring poststress. After the last prenuclear syllable, the prosodic contour down steps progressively until the utterance end. By contrast, the yes/no question demonstrates higher initial peaks aligned with stressed syllable offsets [29, 30]. The observed differences in early pitch movements are reported to play a crucial role in shaping the perception of sentence types in Spanish [19, 31], as well as in other Romance languages such as Italian [32]. An auditory gating experiment conducted with native Spanish speakers, for instance, demonstrated that participants were able to accurately identify the sentence type with a 95% accuracy rate after being exposed to just the contour that included the initial f0 peak [31]. In addition to the previous cues, two less commonly observed intonation patterns also contribute to this perceptual process, albeit to a lesser extent. One is f0 movement in the medial position of the sentence, characterized by a rising f0 pattern in statements that consist of more than two stressed words, a feature not present in yes/no questions. The other is the f0 contour observed during the final stressed syllable, where statements typically present exhibit a slight rise in f0, while yes/no questions tend to show an f0 dip [19, 31]. Despite these observations, the impact of these particular intonation patterns on L2 learners’ perception of sentence modality remains a less explored area. Future research is needed to delve deeper into understanding how these subtleties in intonation might influence native tonal language speakers’ interpretation of sentence modality in Spanish and other Romance languages.

Furthermore, the positioning of the final stressed syllable may modulate Spanish intonation processing by adjusting the shape of the final pitch movement [33, 34]. Prior research indicates that rising-falling patterns are particularly susceptible to the placement of the final stressed syllable [20]. Nevertheless, it remains unclear whether variations in utterance-final word stress affect the perception of Spanish intonation by native and non-native listeners. Experimental studies focusing on this issue found that Mandarin learners of Spanish struggled to recognize words with stress on the last syllable compared to those with stress on the penultimate syllable, potentially due to conflicting functions of f0 in the final position of oxytone words [35, 36]. Yet this study examined L2 learners’ sensitivity to continuous changes in three acoustic dimensions (f0, duration, and intensity) of intonation. Further research is therefore needed to investigate potential impacts of stress patterns on categorical perception of Spanish intonation contours vital for signaling sentence modality in both native and non-native speakers.

### 1.2 Interspeaker variations in L2 perceptual processing

Although speakers within the same linguistic community often exhibit consistent behaviors, significant individual variations can still be observed in processing complex linguistic categories [37, 38]. The impact of speaker-specific factors on speech perception has been interpreted in many ways over time, with numerous dimensions of individual differences being investigated. For instance, research on the relationship between L2 proficiency and speech production/perception has shown mixed results. While some studies have found higher proficiency level correlates with better phonetic and phonological performance in L2 speech production [26, 39–41], the link between proficiency and L2 perception is less clear. One study revealed no significant correlation between Mandarin learners’ L2 proficiency and perceptual accuracy of Spanish questions [25]. However, other research indicates that English learners improve their perception of Spanish prenuclear pitch alignment approaching native-like performance as L2 proficiency increases [26]. High-proficiency adults also appear more accurate at between-category discrimination of non-native novel sounds, potentially due to superior ability to use higher-level cognitive processing like attention to perceive target phonemic boundaries [42, 43].

On the other side, early L2 acquisition is believed to enhance perception yielding more target-like performance versus adult acquisition [4, 14, 42, 43]. For example, early Spanish-English bilinguals show higher accuracy than late bilinguals in categorical discrimination of English vowels [4]. Although some studies have found no significant early versus late bilingual perceptual differences, neural processing distinctions have been observed in L2 sounds, suggesting that the age of acquisition may affect the activation of brain regions necessary for auditory processing [42]. However, compared to the age of acquisition, the effect of listeners’ chronological age on perceiving L2 Spanish phonetic and phonological contrasts has received less attention. Gender is another biological factor, akin to age, that may potentially shape perceptual abilities. Females demonstrate greater accuracy in identifying the prominence of English stressed words compared to males [7]. However, this distinction may be limited to semantic processing and potentially not extend to other processing types [15]. Future research should therefore examine the role of gender in di-verse perceptual tasks like Spanish intonation identification, providing insights into the complex interplay between gender, language context, and sentence modality while elucidating cross-linguistic gender-based perceptual learning strategies.

Psychometric measurements also offer interpretations of individual perceptual differences. Previous studies have linked “autistic traits” per the Autism Spectrum Quotient (AQ) to interspeaker variation in prominence identification [7, 44–46]. For example, English listeners with higher AQ scores (thus poorer pragmatic skills) exhibited lower sensitivity to prosodic manipulation of prenuclear prominence in online lexical processing [7, 44], as well as lower perception of prominence in words parsed into phonologically weak positions [4]. However, as with biological factors like age and gender, psychometric perceptual effects likely depend on the specific linguistic categories being distinguished.

Finally, musical expertise has been theorized to enhance speech perception abilities. For instance, multiple studies have shown that musicians have greater sensitivity to changes in relative pitch structures and melodic contours in both native [47, 48] and non-native languages [49, 50]. Similarly, individuals with music training experience were found to exhibit better L2 pronunciation [51] and excel at discriminating various speech features including phonemic vowel length contrasts [52] and lexical tone variations [53, 54]. However, musical advantages in perception may only apply to auditory tasks relying on the same type of skills developed through long-term music practice, like pitch and duration processing [55–57]. Further research is necessary to determine if L2 learners with advanced musical abilities demonstrate an improved perception of Spanish sentential contrast, given the importance of pitch and melodic processing in music and intonation.

### 1.3 The present study

Building on earlier findings, speech perception emerges as a multifaceted process shaped by factors across dimensions. Comprehensively understanding tonal language speakers’ perception of Spanish sentences necessitates considering both prosodic and individual factors broadly. The goals of this study are to (1) assess the auditory performance of Mandarin learners of Spanish in perceiving broad focus statements and information-seeking yes/no questions, and (2) examine the role of diverse factors in processing L2 sentence modalities. Specifically, we investigate two underexplored research questions for the Mandarin-Spanish language pair:

RQ1: How do intonation contours and final stress placement affect native Mandarin speakers’ perception of Spanish sentence modalities?
RQ2: Is Mandarin speakers’ processing of Spanish question-statement contrasts modulated by individual characteristics including L2 proficiency, age, gender, pragmatic skill, and musical ability?

To address these questions, we constructed a perceptual test drawing upon the gating paradigm as utilized in prior studies [16, 28, 52]. The subsequent sections describe the process of preparing the auditory stimuli and administering the online experiment.

## 2 Methods

### 2.1 Ethics statement

The study was conducted online using an electronic survey. The introductory page of the survey detailed the participation requirements, experimental procedures, and data privacy policy. By clicking ’I agree and next’ at the bottom of this page, participants provided documented confirmation of informed consent, affirming they met the over 18 age criteria and comprehended the instructions. The participation was entirely voluntary, and participants could stop and withdraw at any time. As this study collected data online without physical harm to the participants and anonymized the data, an exemption from full ethics review was granted by the Ethics Committee of Beijing Institute of Technology (approval code: BIT-EC-H-2023132).

### 2.2 Participants

**Fig 1:**
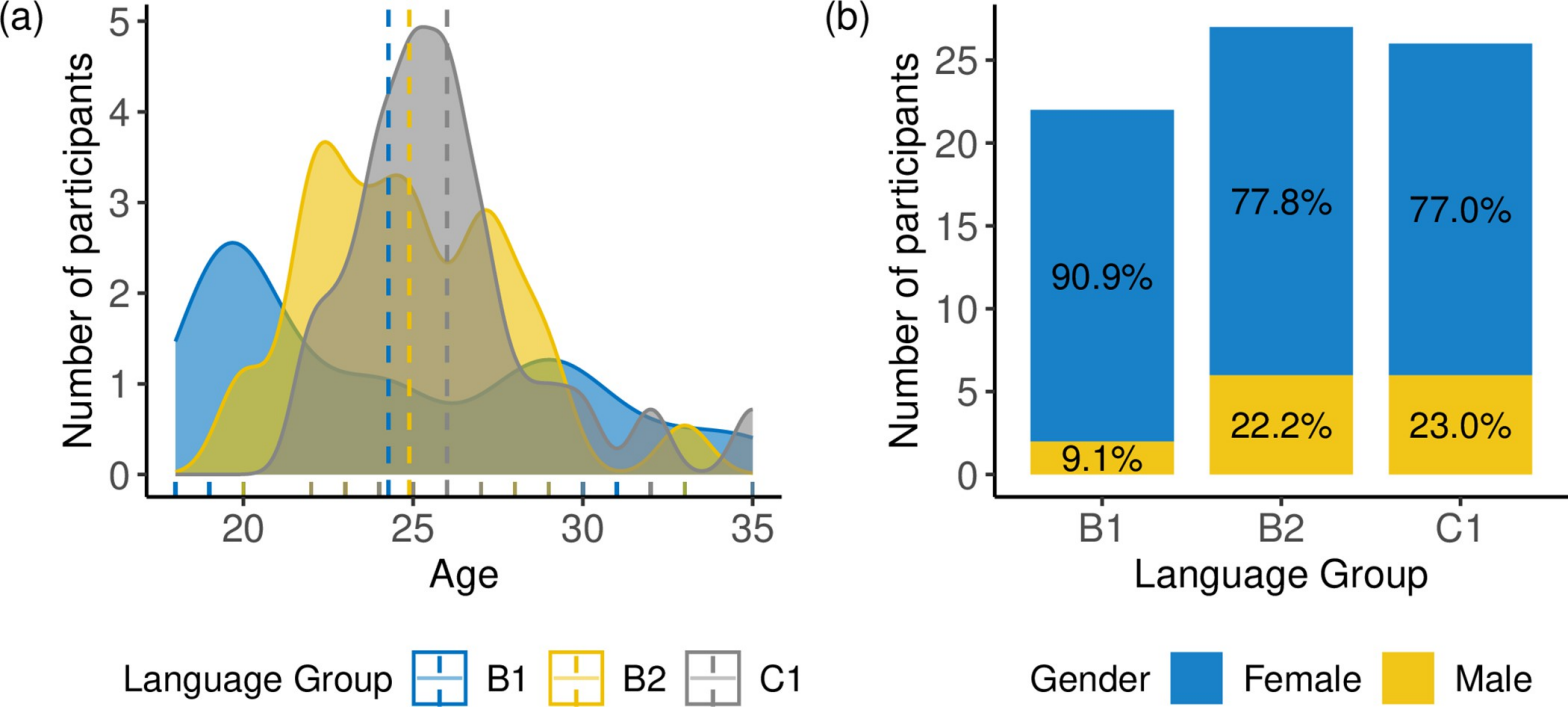
Age and gender distribution of Mandarin participants. (The dotted lines represent mean age values).

The study included a total of seventy-five native Mandarin speakers (*M*_*age*_ = 25.09, *SD* = 3.76, *Min*_*age*_ = 18, *Max*_*age*_ = 35), comprising 28% men and 72% women. Participants were compensated monetarily for their participation in the experiment. All participants were born in mainland China and reported being predominantly exposed to Peninsular Spanish during their language learning process. On average, they began learning L2 Spanish at the age of 19.73 years (*SD* = 3.00). The participants were divided into three language groups based on their proficiency level in Spanish: B1 (*N* = 22), B2 (*N* = 27), and C1 (*N* = 26). While approximately 60% of the participants’ L2 proficiency was assessed using their recent Spanish DELE (Diploma of Spanish as a Foreign Language) certificate, the remaining participants were asked to self-evaluate their Spanish proficiency using the six reference levels in the Common European Framework of Reference for Languages (CEFR). Fig 1 displays the distribution of age and gender in each language group. None of the participants reported any history of hearing or communication problems during testing.

Given the limited number of subjects meeting specific selection criteria and the relatively weaker predictive value of factors such as participants’ place of origin in China, L2 immersion status, and length of exposure to the target language environment for L2 phonological and phonetic accuracy [58, 59], the present study did not strictly control for these variables. However, Table 1 has provided this information to complete the sociodemographic profiles of the participants.

**Table 1:** Mandarin participants’ immersion status and exposure length in Spain.

Immersion status in Spain		Exposure time in Spain				
Yes	No	< 3 months	3 ~ 8 months	6 months ~ 1 year	1 year ~ 2 years	> 2 years
49	26	3	2	11	18	41

### 2.3 Materials

Four pairs of broad focus statements and information-seeking yes/no questions were used to create stimuli. Each pair consisted of a morpho-syntactically identical statement and yes/no question (see Table 2). The four pairs differed in length and therefore in the number of stressed words. For each length, we included two stress patterns for the last word: penultimate stressed syllable (paroxytone) and final stressed syllable (oxytone). Our study utilized the Discourse Completion Task (DCT) [60] method to elicit the semi-spontaneous production of two distinct intonation types, each fulfilling a specific linguistic function: broad focus statements and information-seeking yes/no questions. To supplement the dialogues’ situational framework, we integrated visual stimuli created through OpenAI’s DALL-E, an advanced AI image generation tool [61]. Each scenario within our dialogue series was paired with an image, generated from a descriptive prompt processed by DALL-E (see S1 Appendix for details of the image generation prompts), to visually underscore the context. These images were assessed by the authors to confirm their representational accuracy and unbiased nature. Additionally, in our study, dialogues were consistently initiated by an interlocutor known to the native Spanish speaker, a methodological decision aimed at mitigating potential biases due to power dynamics and social distance [62].

**Table 2:** Categorization of the recorded utterances used to create stimuli.

Sentence modality	No. of stressed words	Stress pattern of the last word	Recordings and English translations
Statement	1	Oxytone	*Alcalá*^a^. [Alcalá.]
Statement	1	Paroxytone	*Sevilla*^b^. [Sevilla.]
Statement	2	Oxytone	*Viene a Alcalá*. [He comes to Alcalá.]
Statement	2	Paroxytone	*Viene a Sevilla*. [He comes to Sevilla.]
Yes/no question	1	Oxytone	*¿Alcalá*? [Alcalá?]
Yes/no question	1	Paroxytone	*¿Sevilla*? [Sevilla?]
Yes/no question	2	Oxytone	*¿Viene a Alcalá*? [Does she come to Alcalá?]
Yes/no question	2	Paroxytone	*¿Viene a Sevilla*? [Does she come to Sevilla?]

^a^Alcalá is the name of a city in the autonomous community of Madrid, Spain, and is pronounced [alkaˈla] in Spanish.

^b^Sevilla is the name of the largest city in the autonomous community of Andalusia, Spain, and is pronounced [seˈβiʎa] in Spanish.

The individual producing the speech material was a 31-year-old female native of Spain, who lived and was educated in urban Cantabria until reaching adulthood. Her intonation represents the standard pattern of Cantabria Spanish, which is consistent with Castilian intonation and epitomizes the most generalized variant of Peninsular Spanish [63–65]. Specifically, her speech production is characterized by a falling contour in broad focus statements and a rising contour in information-seeking yes/no questions. It is crucial, moreover, to distinguish these standard patterns from the traditional variety of Cantabrian Spanish, mainly preserved in the rural areas of northwest Peninsula and marked by a contrasting final falling contour (H* HL%) in yes/no questions [63, 64]. In this study, we did not include this rural pattern because it diverges considerably from most Spanish varieties and is often not included in L2 Spanish instructional materials and settings. The rising pattern for yes/no questions, being more prototypical and widely recognized, was therefore selected as the basis for our stimulus creation, reflecting the intonation most Mandarin learners are likely to be familiar with. For a detailed overview of the elicitation procedure and the sample dialogues, refer to the S1 Appendix. The recordings were conducted in a speech lab using a Rode Smartlav+ microphone connected to a Scarlett interface. The audio files were digitized at a 44.1 kHz sampling rate with 16-bit quantization precision.

### 2.4 Stimulus creation

The recordings described in the Materials section were used to generate stimuli within a gating paradigm. We divided the recorded sentences into several fragments, also referred to as gates, based on intonational events rather than segmental information, which contain intonational differences between the two sentence modalities. As a result, the gates we created align with established intonational events in Spanish phonology, and sentences containing one and two stressed words result in different numbers of gates due to this methodology. Moreover, it is crucial to indicate that, unlike some common practices in the field of auditory gating experiments, which involve manipulating the intonation contours through adjustments to pitch height [19, 31, 66], our procedure focused solely on segmenting the utterances without any modification to the acoustic correlates. This strategy ensured that listeners were gradually exposed to the sentences’ natural speech pattern, preserving the authentic prosodic characteristics of the spoken language.

**Fig 2:**
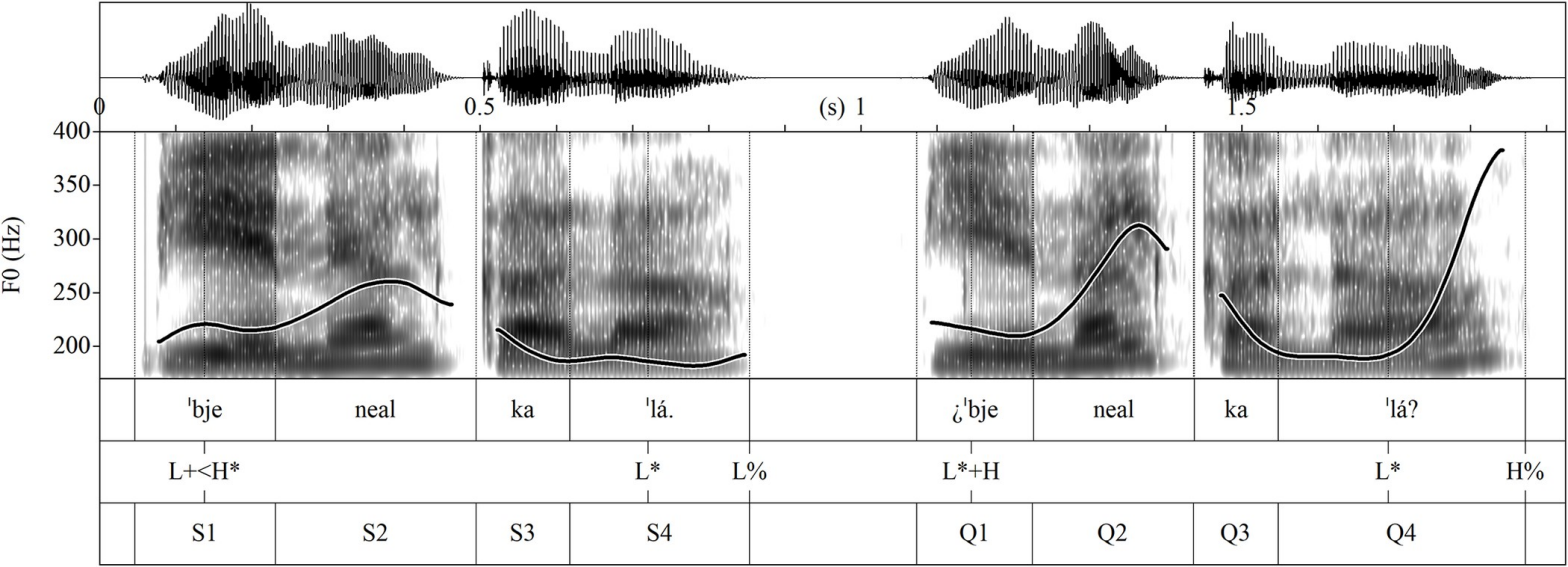
Intonation contours of the sentence pair with two stressed words aligned with syllables and gates. (S1, gate 1 of the statement; Q1, gate 1 of the yes/no question; and so on for the rest of the abbreviations).

For sentences with two stressed words, Gate 1 contained the intonational contour of the first stressed syllable *Vie*, where the statement had a slight rise starting near the beginning of the stressed syllable, while the question did not (see Fig 2). Gate 2 consisted of the initial f0 peak, which may sometimes include an adjacent syllable of the following word due to synalepha. This peak was reported to be the first strong differential cue in determining the type of long sentences because questions have a different prenuclear accent than statements in Spanish [19, 29]. The difference can also be seen in Fig 2 where the yes/no question has a 71 Hz higher f0 peak (335 Hz) than the statement (264 Hz). Gate 3 begins after the first peak and ends before the utterance-final syllable. Therefore, for sentences ending with a paroxytone word, gate 3 included the contour of the final stressed syllable, which unequivocally differentiates the question (rising contour) from the statement (low-falling contour). For sentences ending with an oxytone word, gate 3 did not contain a stressed syllable. This means that gate 3 had more cues for correct recognition in final paroxytone sentences than in oxytone ones. However, we kept this division to have the same number of gates for both stress types and to explore the effect of the final stressed syllable on intonation categorization. The final gate (gate 4) of sentences with two stressed words contained the intonation contour of the last syllable, which is traditionally the last but most typical cue for signaling the question-statement contrast.

**Fig 3:**
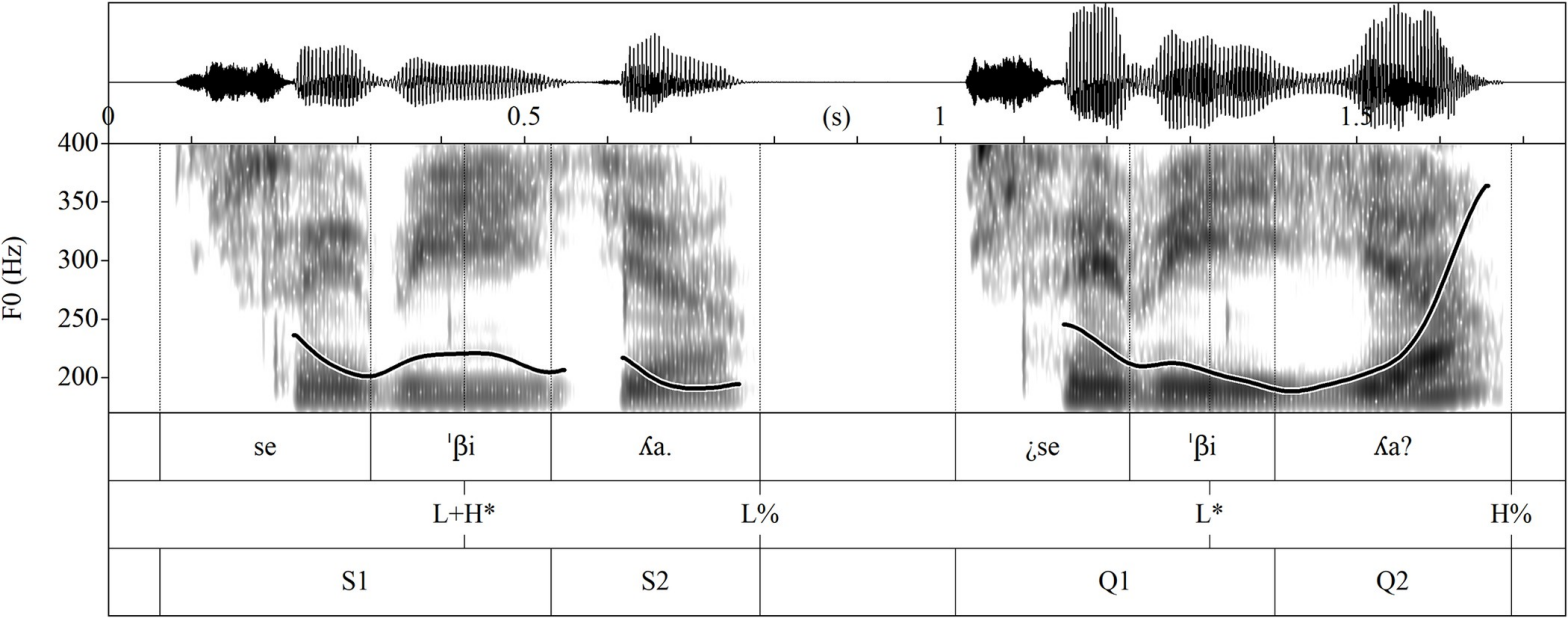
Intonation contours of the sentence pair with one stressed word aligned with syllables and gates.

For sentences comprised of a single stressed word, we created two gates, positioning the division boundary just before the final syllable (see Fig 3). As a result, the prosodic configuration of gate 1 in these single-word sentences exhibited subtle variations based on the stress pattern. In the case of paroxytone words, gate 1 included the contour of the nuclear stressed syllable, which usually yields a relatively high-rising pitch for statements and a low-falling pitch for yes/no questions. Conversely, for oxytone words, we intentionally segmented gate 1 to exclude the nuclear stressed syllable, establishing a distinct perceptual contrast between the two types of stress patterns. Gate 2, the final gate, consisted of the intonation contour of the entire sentence, encompassing the typical final pitch movements observed in both statements and questions.

The segmentation of speech was performed using Praat [67]. To create the final stimuli for the experiment, we progressively compiled the gates, starting with the first gate alone, followed by a combination of the first and second gates, and continuing in this manner until the entire utterance was incorporated. This process resulted in a total of 24 distinct stimuli for the experiment. Each stimulus was saved as a separate sound file, with an added 500 milliseconds of silence both preceding and following the spoken content. Audio clips for the stimuli generated in this study are available in the S2 Appendix.

### 2.5 Procedure

The Alchemer survey platform (https://www.alchemer.com/) was employed for data collection. The survey comprised four parts. The first was designed to gather sociolinguistic information including the participant’s origin, age, gender, education, and linguistic background such as L2 proficiency, L2 immersion status, exposure time in Spain, and so forth.

The second part focused on measuring participants’ musical ability, which pertains to their sensitivity in perceiving various auditory features such as pitch, melody, tuning, rhythm, and tempo. Particularly, this study aims to measure participants’ perceptual musical abilities to pitch and melody, which are considered primary phonetic properties of sentential intonation in Spanish [68]. To achieve this, we employed two specific tasks: the pitch and melody subsets from the PROMS (Profile of Music Perception Skills) test [69]. During both tasks, participants were presented with audio pairs of different complexities via earphones and were required to rate the similarity of the test sound to the reference audio clip using a five-point scale (*definitely the same*, *probably the same*, *I don*’*t know*, *probably different*, *definitely different*). The final scores for each participant were automatically generated and subsequently gathered by the first author.

The third part aimed to assess participants’ pragmatic skills and included items from the Autism Spectrum Quotient (AQ) questionnaire. The complete AQ contained five subscales: social skills, communication, attention switching, attention to details, and imagination [70]. In this study, we selected the 10 tokens from the communication subscale, which have been considered a rough proxy for speakers’ pragmatic ability to engage the prosody in a specific context [7]. Participants’ responses to these items were evaluated with a four-point scale (*strongly agree*, *slightly agree*, *slightly disagree*, *strongly disagree*) and their total AQ scores were calculated by summing the score for each. Higher scores on the AQ indicated more “autistic-like” traits and therefore were considered to reflect poorer pragmatic skills [71]. The original English AQ items were translated into Chinese based on prior work [72].

Finally, the fourth part presented the stimuli for the perceptual test, during which participants were instructed to listen to each of them using earphones in a quiet room. Before beginning the formal task, participants completed a practice trial to familiarize themselves with the procedure. The full text of the audio was displayed on the screen without punctuation marks. The task of the participants was to identify whether the stimulus they heard was a “Statement” or a “Yes/no question” by clicking the appropriate button on the screen. The perceptual choices and stimuli were presented in a random order to avoid listeners’ response bias.

### 2.6 Statistical analysis

Before statistical modeling, compound musical ability scores were computed for each participant by averaging the pitch and melody task scores. Then, we performed a two-step cluster analysis of these scores to automatically classify the participants into high and low musical ability levels. Generalized linear models (GLMs) subsequently were used to analyze the dataset, with perceptual accuracy (true vs false) as the dependent variable operationalized by participant responses to each stimulus. The GLMs incorporated binary variables including gender (male vs. female), sentence modality of the stimulus (statement vs. yes/no question), stress position of the final word (final vs. penultimate), and musical ability (high vs. low). Discrete ordinal variables included L2 proficiency level (B1 < B2 < C1) and gate (1 < 2 < 3 < 4 for longer sentences; 1 < 2 for shorter sentences). Meanwhile, age, AQ score, and stimulus order were treated as continuous factors and standardized via z-transformation prior to model inclusion.

Two multivariable GLMs were constructed in this study, one for sentences with one stressed word and one for sentences with two stressed words. Stepwise backward elimination was performed using the step AIC function from the MASS package to determine the best-performing models [73]. Additionally, EMMs objects were generated with the contrast function to analyze model interactions [74]. The R software environment was utilized for all statistical analyses [75].

## 3 Results

### 3.1 Results for sentences with two stressed words

The significance test of the first GLM revealed that Mandarin learners’ accuracy in perceiving sentences with two stressed words was significantly influenced by the interaction between sentence modality and gate [χ^2^(3) = 24.25, *p* < .0001], L2 proficiency [χ^2^(2) = 14.09, *p* < .001], pragmatic skill [χ^2^(1) = 31.19, *p* < .0001)], and musical ability [χ^2^(1) = 6.04, *p* < .05]. However, stress position, age, and gender did not significantly impact auditory evaluation of L2 sentences. Regarding the interaction of sentence modality with gate, Table 3 shows that Mandarin learners performed significantly better identifying L2 statements than yes/no questions without salient interrogative cues. Particularly, the contrast coefficient, transformed into odds ratios (OR), indicated that statements were 4.03 times more likely to be correctly recognized by Mandarin learners in gate 1 relative to yes/no questions.

**Table 3:** Results of the regression model fitted to sentences with two stressed words. (The estimated coefficients and confidence intervals were transformed from log-odds to odds ratios).

Predictors	Estimates	95% CI	SE	*z value*	p
(Intercept)	0.28	[0.17, 0.46]	0.26	-4.95	< .001***
Sentence modality*Gate (S1^a^ vs. Q1^b^)	4.03	[2.46, 6.63]	0.25	5.51	< .001***
Sentence modality*Gate (S2 vs. Q2)	0.73	[0.44, 1.19]	0.25	-1.26	0.90
Sentence modality*Gate (S3 vs. Q3)	1.27	[0.69, 2.34]	0.31	0.77	0.99
Sentence modality*Gate (S4 vs. Q4)	4.11	[0.45, 37.34]	1.13	1.26	0.90
Stress position (Penultimate vs. Final)	0.94	[0.70, 1.27]	0.15	-0.38	0.70
Proficiency (B2 vs. B1)	1.70	[1.16, 2.48]	0.19	2.73	< .01**
Proficiency (C1 vs. B1)	2.10	[1.41, 3.14]	0.20	3.67	< .001***
Proficiency (C1 vs. B2)	1.24	[0.86, 1.79]	0.19	1.16	0.48
Pragmatic skill (AQ)	0.63	[0.54, 0.74]	0.08	-5.58	< .001***
Musical ability (High vs. Low)	1.56	[1.09, 2.23]	0.18	2.46	< .05*
Age	1.23	[0.88, 1.73]	0.17	1.21	0.23
Gender (Male vs. Female)	0.89	[0.60, 1.31]	0.20	-0.60	0.55

^a^S1, gate 1 of the statement.

^b^Q1, gate 1 of the yes/no question; and so on for the rest of the abbreviations.

**Fig 4:**
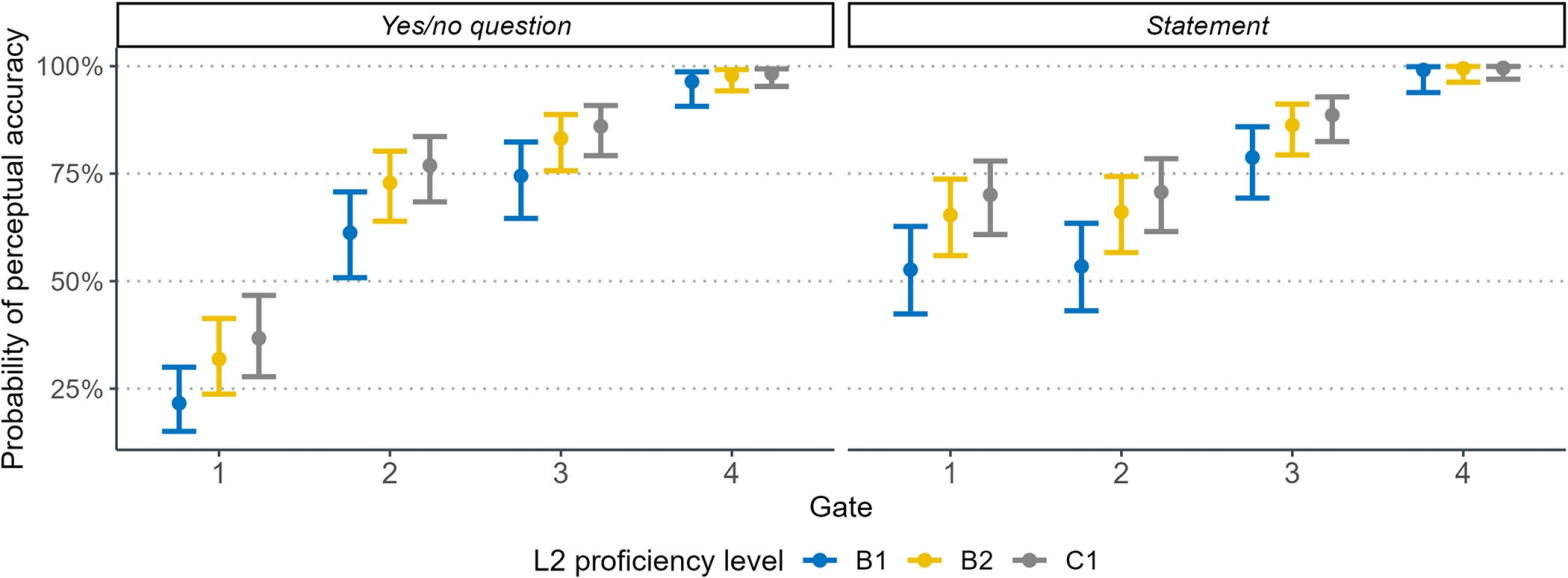
Effects of L2 proficiency level on perceiving sentences with two stressed words at each category of sentence modality and gate.

Table 3 also reveals that Mandarin learners’ ability to perceive Spanish sentential contrasts is associated with their proficiency level in L2. As depicted in Fig 4, learners with higher proficiency levels (e.g., B2 and C1) displayed significantly greater accuracy in distinguishing L2 sentence modalities compared to those with a B1 level. Although the interaction between L2 proficiency and gate did not reach statistical significance, multiple comparisons revealed distinct auditory performance among the three language groups after exposure to each of the four gates. Specifically, from gate 1 to gate 2, both B2 (z = 3.08, *p* < .05) and C1 learners (z = 3.54, *p* < .01) exhibited significant progress in perceptual accuracy, while B1 learners did not improve evidently after perceiving the initial f0 peak (gate 2). B1 and B2 learners then significantly increased perceptual accuracy (both *ps* < .05) after being presented with the f0 downslope after the highest peak (gate 3). Finally, the three L2 groups neared 100% accuracy upon the release of the final f0 contour, but only B1 learners significantly improved after listening to the last gate.

**Fig 5:**
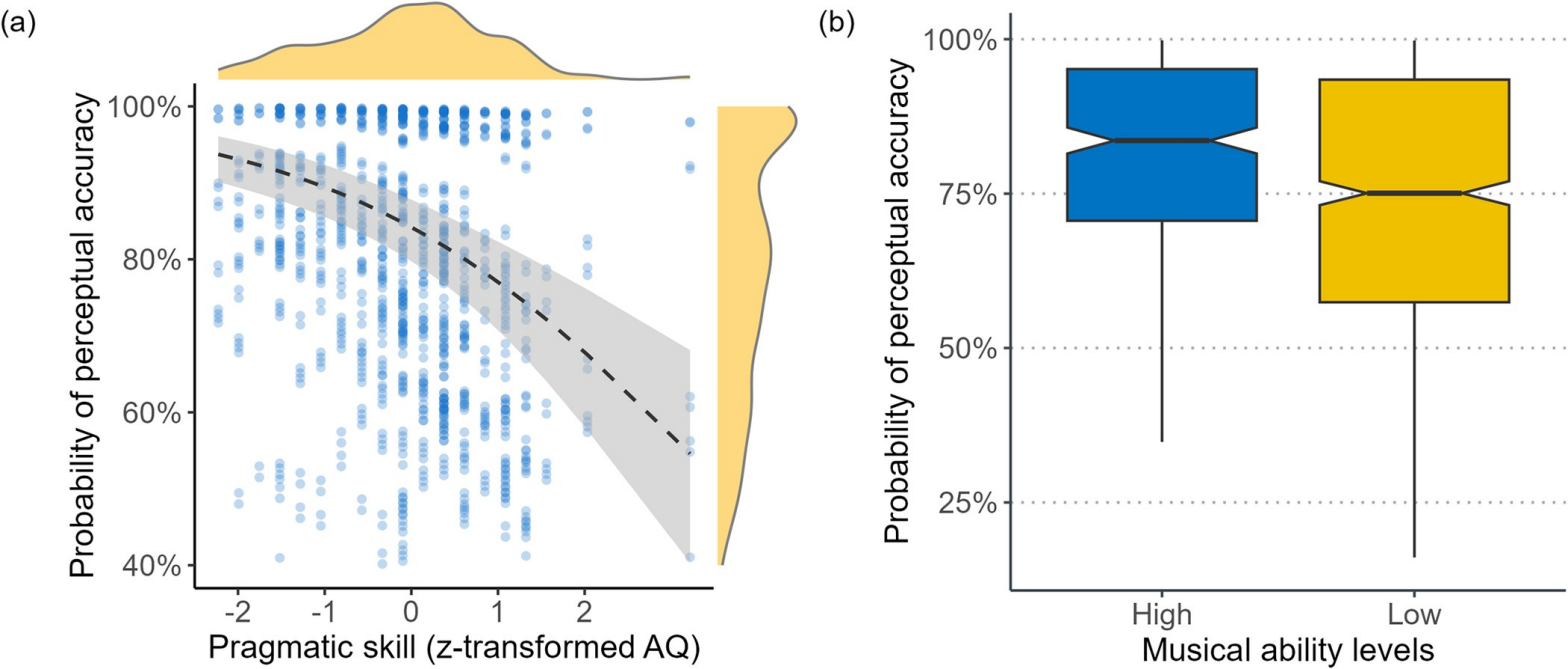
Effects of pragmatic skill (a) and musical ability (b) on perceiving sentences with two stressed words.

Additionally, the results presented in Fig 5A show a robust negative correlation between the pragmatic skills of Mandarin learners, as measured by the AQ score, and their ability to accurately associate intonation patterns with sentence modalities in L2 Spanish. This association is further supported by the coefficient of the AQ variable, as indicated in Table 3, which suggests that for every one standard deviation increase in the AQ score, the likelihood of correctly identifying L2 Spanish sentence types decreases by 37%. Further, the results depicted in Fig 5B indicate that Mandarin learners with higher levels of musical ability performed significantly better in perceiving L2 sentential contrasts. Specifically, individuals with high musical ability levels exhibit a 56% higher likelihood of accurately distinguishing Spanish sentence modalities compared to those with lower musical ability levels.

### 3.2 Results for sentences with one stressed word

The significance test of the second GLM indicated that the L2 perception of Spanish sentences with one stressed word was significantly impacted by sentence modality [χ^2^(1) = 52.60, *p* < .0001], gate [χ^2^(1) = 70.39, *p* < .0001], stress pattern [χ^2^(1) = 4.77, *p* < .05], pragmatic skill [χ^2^(1) = 15.33, *p* < .0001], and musical ability [χ^2^(1) = 4.36, *p* < .05]. However, L2 proficiency, age, and gender did not significantly influence one-word sentence perception (all *ps* > .1).

**Fig 6:**
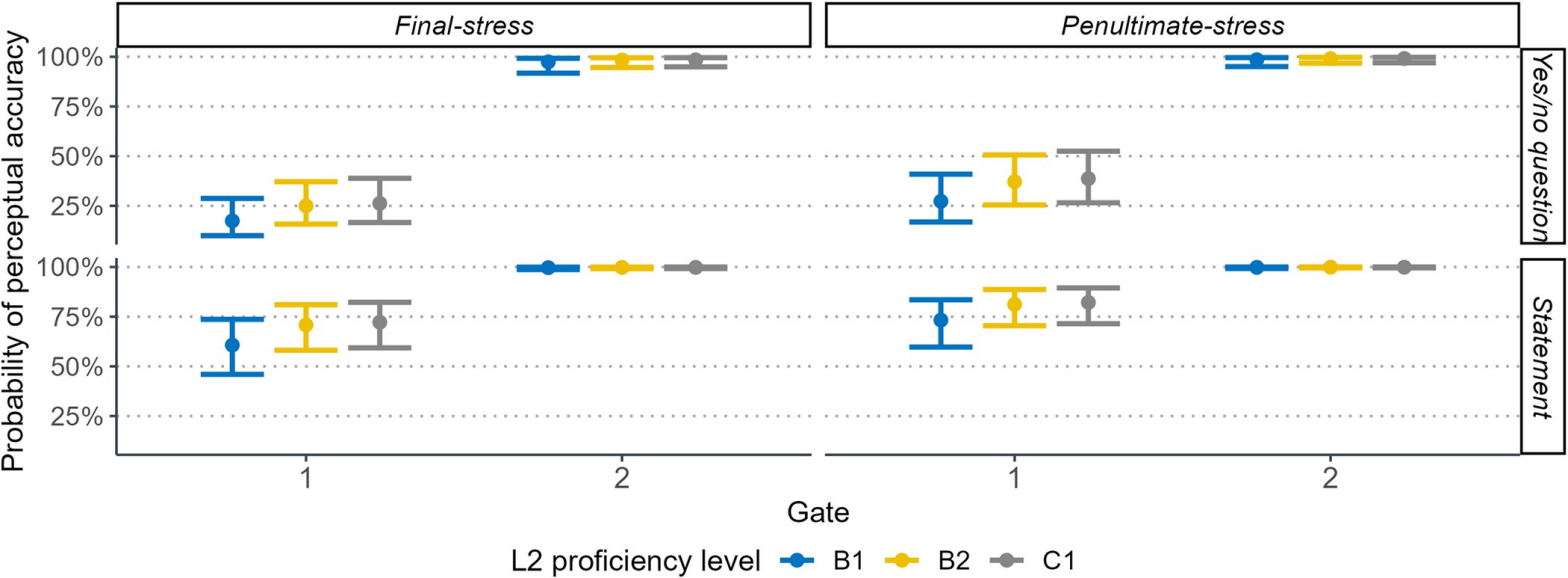
Effects of L2 proficiency level on perceiving sentences with one stressed word at each category of sentence modality, gate, and stress position.

Table 4 shows that statements were significantly more likely to be correctly identified than yes/no questions, particularly in gate 1 (see Fig 6). All three language groups exhibited a significant increase in perceptual accuracy from gate 1 to gate 2. Likewise, the effect of stress position indicated that paroxytone words were easier for learners to perceive compared to oxytone words. This distinction was primarily observed in gate 1 of the two types of stressed words (z = -2.37, *p* < .05), while in gate 2, the difference did not attain statistical significance (z = 0.57, *p* > .1).

**Table 4:** Results of the regression model fitted to sentences with one stressed word. (The estimated coefficients and confidence intervals were transformed from log-odds to odds ratios).

Predictors	Estimates	95% CI	SE	*z value*	p
(Intercept)	0.19	[0.09, 0.39]	0.37	-4.50	< .001***
Sentence modality (St^a^ vs. Qu^b^)	7.29	[53.53, 605.75]	0.27	7.25	< .001***
Gate (2 vs.1)	180.07	[0.44, 1.19]	0.62	8.39	< .001***
Stress position (Penultimate vs. Final)	1.77	[1.06, 2.97]	0.26	2.18	< .05*
Proficiency (B2 vs. B1)	1.58	[0.82, 3.05]	0.34	1.35	> .1
Proficiency (C1 vs. B1)	1.68	[0.86, 3.30]	0.34	1.51	> .1
Proficiency (C1 vs. B2)	1.07	[0.58, 1.97]	0.31	0.21	> .1
Musical ability (High vs. Low)	1.88	[1.04, 3.41]	0.30	2.09	< .05*
Pragmatic skill (AQ)	0.57	[0.43, 0.76]	0.14	-3.92	< .001***
Age	1.02	[0.57, 1.83]	0.30	0.06	> .1
Gender (Male vs. Female)	0.81	[0.42, 1.58]	0.34	-0.61	> .1

^a^ St, statement.

^b^ Qu, yes/no question.

Furthermore, results in Table 4 showed that the impact of pragmatic skills on perceiving one-word sentences aligned with previous findings for longer sentences, with a 43% lower likelihood of perceptual accuracy per standard deviation increase in AQ score (Table 4). Similarly, learners with higher musical ability were found to relate to 1.88 times greater accuracy in identifying Spanish sentence modality versus those with inferior musical ability. Fig 6 also illustrates that Mandarin learners were able to enhance their identification rates as L2 proficiency improved. However, this enhancement was not statistically significant in perceiving sentences with one stressed word, which seems to contradict previous results for longer sentences. This discrepancy likely stems from the distribution of intonation cues across one-word stimuli. Specifically, in gate 1, all three L2 groups displayed similarly low perceptual accuracy due to the lack of robust cues that clearly indicated sentence modality. In contrast, gate 2 provided the salient cue of the final pitch contour universally signaling question-statement contrast, enabling perception improvements regardless of Mandarin learners’ proficiency in Spanish.

## 4 Discussion

The current study formulated two research questions aimed at exploring the cross-linguistic perception of Spanish sentence modalities by Mandarin-speaking listeners. The first focused on examining the influence of prosodic components, including intonation contours and the final stress pattern, on perceiving Spanish broad focus statements and information-seeking yes/no questions. The results demonstrate the pivotal role of prosodic elements in determining question-statement identification in a cross-linguistic context. However, the efficacy of these two factors appears to vary based on sentence modality, L2 proficiency, and utterance length. Particularly, our study found that Mandarin learners were more inclined to perceive an utterance as a statement in gate 1 where no salient interrogative cues were present. This observation aligns with prior studies involving native Spanish listeners [19, 25, 31, 76], suggesting a universal inclination towards unmarked linguistic forms in communication. Declaratives are generally considered the most neutral and unmarked sentence type, carrying the least communicative load across languages including Spanish, Basque, and various Germanic languages [77–79]. Consequently, their default manifestation in the form of statements would be more readily processed and understood than yes/no questions by Mandarin learners of Spanish. This propensity underscores the cognitive efficiency of utilizing unmarked structures in language comprehension and acquisition. Our results also reveal that all intonational cues distributed across the utterance contributed somewhat to identifying Spanish sentences, but the ability to accurately recognize these cues depended on learners’ L2 proficiency. Highly proficient Mandarin learners at the B2 and C1 levels exhibited significant improvements in identifying sentences by utilizing the initial f0 peak, while B1 learners struggled to use this information to signal question-statement contrasts. Similar challenges in L2 Spanish perception were reported in past work by Trimble [76] and Li [25], potentially stemming from insufficient experience with target prosody.

Moreover, the results showed that learners at the lower proficiency level (B1) relied primarily on the f0 downslope of the nuclear syllable and final pitch movement to identify sentences containing two stressed words. In contrast, the final intonational pattern did not seem to significantly impact the identification of longer sentences for learners at the C1 level. However, further analysis using single-word utterances revealed the final pitch shift as the most reliable cue for all L2 learners in perceiving question-statement contrasts, once acoustic interference from the initial f0 peak and the nuclear syllable contour was excluded. These findings are consistent with previous research, which reported the final pitch contour as the strongest cue in discriminating Spanish sentence modality, capable of changing native listeners’ perceptual decisions made with earlier contradictory cues [19]. The consistency in auditory weighting for the final intonational contour between Spanish L1 and Mandarin L2 speakers suggests potential universal effects on specific intonational forms and their paralinguistic meanings in speech communication [27, 80, 81].

Our research further revealed that the impact of the final stress position on the perception of L2 Spanish sentence modalities is modulated by utterance length. Notably, in short sentences, we observed a significantly higher perceptual accuracy for penultimate stressed words than final stressed words, particularly when identified within gate 1. This disparity is mainly ascribed to the pitch movement contrasts present in gate 1 of the penultimate stressed words, wherein the nuclear stressed syllable typically displayed a high-rising contour in statements and a divergent low-falling contour in yes/no questions. Our data suggests that Mandarin learners were sensitive to these phonetic distinctions, utilizing them as an important cue to discern target sentence modalities, thereby improving the perception of paroxytone words. In contrast, long sentences containing two stressed words and ending with a paroxytone manifested a uniform low-falling contour on the final stressed syllable across both statements and yes/no questions, eliminating a distinctive phonetic feature that could aid in long sentence identification. Consequently, we observed no significant variance in perceptual accuracy for long sentences ending with diverse stress patterns. These findings lend support to prior research which asserts that the pitch contour of the final stressed syllable is a weaker cue for the discrimination of long sentences in Spanish, yet it remains pivotal for assessing the naturalness of interrogative sentences [31].

Regarding the second research question, our results indicated that individual characteristics such as L2 proficiency, pragmatic skills, and musical ability can fine-tune Mandarin learners’ auditory processing and cognitive abilities, thereby impacting their perceptual performance in L2. Highly proficient Mandarin learners were more accurate at identifying Spanish statements and questions, especially pitch-contrastive structures not present in their L1 tonal categories, e.g., the initial f0 peak. This finding corroborates previous studies linking enhanced L2 proficiency to improved cross-linguistic perception, including speech comprehensibility [82] and speech recognition in noise [83]. A potential explanation is that high proficiency correlates with increased activity in later-developing brain regions related to higher-order cognition like attention, which may aid in processing non-native speech objects [14].

Another individual feature significantly affecting L2 sentence perception is the pragmatic skill. Our study found that Mandarin learners with poorer pragmatic skills, as indexed by higher AQ scores, were less adept at recognizing the mapping relationship between intonational patterns and linguistic meanings in specific contexts. This aligns with previous research on psychometric properties in speech production and perception [7, 44–46], which, for example, found that English listeners with lower pragmatic skills had greater difficulty perceiving prominence patterns in their L1 as well. Although the precise mechanisms underlying individual variations in pragmatic ability remain unclear, there appears to be a relationship between pragmatics and prosodic sensitivity.

The last individual factor important to the perception of L2 sentence modality consists of musical ability. Our results showed that Mandarin learners with strong musical abilities performed better in perceptual processing of Spanish question-statement contrasts compared to those with low musical abilities. This corroborates previous work on music-language interaction, which demonstrates that high levels of music performance skills can facilitate speakers’ learning process in multiple aspects of speech perception, including lexical tones [18] and melodic contours [47]. This advantage likely stems from a positive transfer of auditory skills developed through music to speech perception, since both domains rely on shared acoustic and neural resources for processing speech signals [56, 84]. Notably, however, we did not find significant effects of biological factors like age and gender on L2 perception of Spanish sentence modality.

## 5 Conclusions

The present study examines the factors impacting native Mandarin speakers’ perception of Spanish sentence modalities. While prior cross-linguistic research between tonal and non-tonal languages has predominantly focused on the role of L1, our results demonstrate that L2 sentence perception entails a more intricate integration mechanism that requires integrating both prosodic features and individual traits. This enables a more comprehensive and precise assessment of L2 perceptual learning outcomes. Furthermore, our findings suggest that the development of sentence type perception toward a more native-like system depends on multifaceted factors, including prosodic awareness, L2 proficiency level, pragmatic skills, and musical ability, potentially acquired through natural linguistic environments or specialized target training. Overall, this study offers valuable insights into the perception of Spanish sentence modalities within a cross-linguistic context and furnishes foundational data that can inform subsequent research aimed at devising training methodologies and techniques for L2 Spanish intonation.

However, some limitations should be acknowledged. First, the gender imbalance among participants may affect the generalizability of our results; hence, future studies should aim for a more balanced representation. Second, the current study’s scope, limited by the range of sentence modalities and the reliance on a single speaker’s speech materials, may not fully capture the spectrum of Spanish intonation patterns. Future research should endeavor to broaden the corpus to include a wide array of native speakers, particularly from the central Castilian region. Such an approach would yield a more comprehensive and balanced investigation that carefully considers both the richness and representativeness of intonation data, alongside the diverse qualities of L2 learners. Third, while our findings suggest that the location of the final stressed syllable affects the L2 sentence perception, the intricate process of how Mandarin learners of Spanish parallelly perceive and decode stress and intonation, particularly when these prosodic features are conveyed through shared acoustic dimensions, remains an area for additional exploration. Moreover, the 10-item AQ communication subscale may not have captured the full complexity of pragmatic competence. Future studies should consider employing a more nuanced array of assessment tools, possibly with multiple raters, to better evaluate this aspect. Lastly, the potential influence of multilingual backgrounds on L2 perception was not controlled for in this study, which is a limitation considering the impact that proficiency in additional languages could have. We plan to make this a key consideration in the design of our future research to ensure a more thorough analysis of the factors affecting L2 sentence perception.

## Supporting information

S1 Appendix Elicitation of corpus. (PPTX)

S2 Appendix Audio files. (ZIP)

S3 Appendix Autism Spectrum Quotient (AQ) items. (PDF)

## References

[1] Shang P, Elvira-García W. Second language acquisition of Spanish prosody by Chinese speakers: Nuclear contours and pitch characteristics. Vigo Int J Appl Linguist. 2022; 129–176. 10.35869/vial.v0i19.3762

[2] Shang P. Cross-Linguistic Comparison of the Pitch and Temporal Profiles between L1 and Chinese L2 Speakers of Spanish. Loquens. 2022; 9: e086–e086. 10.3989/loquens.2022.e086

[3] Shang P. Adquisición de la entonación de las oraciones interrogativas del español por parte de los sinohablantes: Patrones entonativos y propiedades fonético-acústicas. Doctoral Dissertation, Universitat de Barcelona. 2022. http://hdl.handle.net/2445/191301

[4] Baigorri M, Campanelli L, Levy ES. Perception of American–English Vowels by Early and Late Spanish–English Bilinguals. Lang Speech. 2019; 62: 681–700. doi: 10.1177/0023830918806933 30354920 PMC6561833

[5] Lee G, Shin DJ, Garcia MTM. Perception of lexical stress and sentence focus by Korean-speaking and Spanish-speaking L2 learners of English. Language Sciences. 2019; 72: 36–49. 10.1016/j.langsci.2019.01.002

[6] Meng Y, Zhang J, Liu S, Wu C. Influence of different acoustic cues in L1 lexical tone on the perception of L2 lexical stress using principal component analysis: an ERP study. Exp Brain Res. 2020; 238: 1489–1498. doi: 10.1007/s00221-020-05823-w 32435921

[7] Bishop J, Kuo G, Kim B. Phonology, phonetics, and signal-extrinsic factors in the perception of prosodic prominence: Evidence from Rapid Prosody Transcription. J Phon. 2020; 82: 100977. 10.1016/j.wocn.2020.100977

[8] Luchkina T, Cole JS. Perception of Word-level Prominence in Free Word Order Language Discourse. Lang Speech. 2021; 64: 381–412. doi: 10.1177/0023830919884089 31778096

[9] Ortega-Llebaria M, Wu Z. Chinese-English Speakers’ Perception of Pitch in Their Non-Tonal Language: Reinterpreting English as a Tonal-Like Language. Lang Speech. 2021; 64: 467–487. doi: 10.1177/0023830919894606 31898931

[10] Ortega-Llebaria M, Nemogá M, Presson N. Long-term experience with a tonal language shapes the perception of intonation in English words: How Chinese-English bilinguals perceive “Rose?” vs. “Rose.” Bilingualism. 2017; 20: 367–383. 10.1017/S1366728915000723

[11] Grabe E, Rosner BS, García-Albea JE, Zhou X. Perception of English Intonation by English, Spanish, and Chinese Listeners. Lang Speech. 2003; 46: 375–401. doi: 10.1177/00238309030460040201 15198113

[12] Zhang S, Li K, Lo WK, Meng H. Perception of English suprasegmental features by non-native Chinese learners. 5th international conference on Speech Prosody. Chicago, USA; 2010. pp. 1–4.

[13] Liu C, Rodriguez A. Categorical perception of intonation contrasts: Effects of listeners’ language background. J Acoust Soc Am. 2012; 131: EL427–EL433. doi: 10.1121/1.4710836 22713017

[14] Richards et al. Age of acquisition and proficiency in a second language independently influence the perception of non-native speech. Physiol Behav. 2018; 176: 139–148. 10.1017/S1366728911000125 PMC6124681 30197550

[15] van Kesteren MTR, Wiersinga-Post JEC. Auditory temporal-order thresholds show no gender differences. Restor Neurol Neurosci. 2007; 25: 119–122. . 17726270

[16] Jiang J, Liu F, Wan X. Perception of melodic contour and intonation in autism spectrum disorder: Evidence from Mandarin speakers. J Autism Dev Disord. 2015; 45: 2067–2075. doi: 10.1007/s10803-015-2370-4 25636678

[17] Bidelman GM, Hutka S, Moreno S. Tone language speakers and musicians share enhanced perceptual and cognitive abilities for musical pitch: evidence for bidirectionality between the domains of language and music. PLoS One. 2013; 8: e60676. doi: 10.1371/journal.pone.0060676 23565267 PMC3614545

[18] Wiener S, Bradley ED. Harnessing the musician advantage: Short-term musical training affects non-native cue weighting of linguistic pitch. Language Teaching Research. 2020. https://doi.org/10.1177%2F1362168820971791

[19] Face TL. The role of intonational cues in the perception of declaratives and absolute interrogatives in Castilian Spanish. Estudios de fonética experimental. 2007; 16: 186–225.

[20] Garrido Almiñana JM. Modelling Spanish intonation for text-to-speech applications. Doctoral Dissertation, Universitat Autònoma de Barcelona. 2008. https://www.tdx.cat/handle/10803/4885

[21] Navarro Tomás T. Manual de entonación española. Madrid: Ediciones Guadarrama Madrid; 1974.

[22] Antonio Quilis. Tratado de fonología y fonética españolas. Madrid: Gredos; 1993.

[23] Shang P, Elvira-García W, Roseao P. La modalidad interrogativa en español y en chino: un enfoque funcionalista. Círculo de Lingüística Aplicada a la Comunicación. 2021; 88: 235–254. 10.5209/clac.78313

[24] Face TL. The intonation of absolute interrogatives in Castilian Spanish. Southwest Journal of Linguistics. 2004; 23: 65–80.

[25] Li W. Producción y percepción de la entonación del español por los sinohablantes: patrones entonativos prototípicos de la declarativa neutra, la interrogativa absoluta neutra y la declarativa con foco estrecho. Doctoral Dissertation, Universitat Autónoma de Barcelona. 2020.

[26] Zarate-Sandez GA, Morales-Front A, Sanz C. Perception and production of intonation among English-Spanish bilingual speakers at different proficiency levels. Doctoral Dissertation, Georgetown University. 2015.

[27] Gussenhoven C. The phonology of tone and intonation. Cambridge University Press; 2004.

[28] Ohala JJ. The frequency code underlies the sound-symbolic use of voice pitch. In: Hinton L, Nichols J, Ohala JJ, editors. Sound symbolism. 1994. pp. 325–347. 10.1017/CBO9780511751806.022

[29] Estebas-Vilaplana E, Prieto P. Castilian Spanish intonation. In: Prieto P, Roseano P, editors. Transcription of intonation of the Spanish language. 2010. pp. 17–48. 10.1515/shll-2012-1125

[30] Hualde JI, Prieto P. Intonational variation in Spanish: European and American varieties. In: Frota S., Prieto P, editors. Intonation in romance. 2015. pp. 350–391. 10.1093/acprof:oso/9780199685332.003.0010.

[31] Face TL. F0 Peak Height and the Perception of Sentence Type in Castilian Spanish. Revista Internacional de Lingüística Iberoamericana. 2005; 3: 49–65. https://www.jstor.org/stable/4167811

[32] Romano A. Rising-Falling Contours in Speech: A Metaphor of Tension-Resolution Schemes in European Musical Traditions? Evidence from Regional Varieties of Italian. In: McKevitt P, Nualláin SÓ, Mulvihill C, editors. Language, Vision and Music (Advances in Consciousness Research). Amsterdam: John Benjamins; 2002. pp. 325–337. 10.1075/aicr.35.30rom

[33] Navarro-Tomás T. Manual de pronunciación española. Publicaciones de la Revista de Filologia Española i. 1974; 3. https://catalogosiidca.csuca.org/Record/UCR.000344815

[34] Fant L. Estructura informativa en español. Estudio sintáctico y entonativo. Upsala: Acta Universitatis Upsaliensis. 1984.

[35] Shang P, Elvira-García W, Li X. Cue weighting differences in perception of Spanish sentence types between native listeners of Chinese and Spanish. Proceedings of 11th International Conference of Speech Prosody. 2022; 644–648. 10.21437/SpeechProsody.2022-131

[36] Shang P, Roseano P, Elvira-García W. Dynamic multi-cue weighting in the perception of Spanish intonation: Differences between tonal and non-tonal language listeners. J Phon. 2024; 102: 101294. 10.1016/j.wocn.2023.101294

[37] Fuchs S, Pape D, Petrone C, Perrier P. Individual differences in speech production and perception. Berlin: Peter Lang. 2015. doi: 10.3726/978-3-653-05777-5

[38] Shang P, Zeng M. The role of native language experience and individual features in the cross-linguistic perception of Spanish intonation. Poster presented at the 10th International Symposium on the Acquisition of Second Language Speech. 2022.

[39] Zhang J, Lee S. Acquisition of English speech rhythm by Chinese learners of English at different English proficiency levels. Phonetics and Speech Sciences. 2019; 11: 71–79. doi: 10.13064/ksss.2019.11.4.071

[40] Li A, Post B. L2 acquisition of prosodic properties of speech rhythm. Stud Second Lang Acquis. 2014; 36: 223–255. 10.1017/S0272263113000752

[41] Graham C, Post B. Second language acquisition of intonation: Peak alignment in American English. J Phon. 2018; 66. 10.1016/j.wocn.2017.08.002

[42] Archila-Suerte P, Zevin J, Hernandez AE. The effect of age of acquisition, socioeducational status, and proficiency on the neural processing of second language speech sounds. Brain Lang. 2015; 141: 35–49. doi: 10.1016/j.bandl.2014.11.005 25528287 PMC5956909

[43] Archila-Suerte P, Zevin J, Bunta F, Hernandez AE. Age of acquisition and proficiency in a second language independently influence the perception of non-native speech. Bilingualism: Language and Cognition. 2012; 15: 190–201. doi: 10.1017/S1366728911000125 30197550 PMC6124681

[44] Bishop J. Focus projection and prenuclear accents: Evidence from lexical processing. Lang Cogn Neurosci. 2017; 32: 236–253. 10.1080/23273798.2016.1246745

[45] Jun SA, Bishop J. Priming implicit prosody: Prosodic boundaries and individual differences. Lang Speech. 2015; 58: 459–473. doi: 10.1177/0023830914563368 27483740

[46] Bishop J. Focus, prosody, and individual differences in “autistic” traits: Evidence from cross-modal semantic priming. UCLA Working Papers in Phonetics. 2012; 111: 1–26.

[47] Fujioka T, Trainor LJ, Ross B, Kakigi R, Pantev C. Musical training enhances automatic encoding of melodic contour and interval structure. J Cogn Neurosci. 2004; 16: 1010–1021. doi: 10.1162/0898929041502706 15298788

[48] Schön D, Magne C, Besson M. The music of speech: Music facilitates pitch processing in language. Psychophysiology. 2004; 41: 341–349. 10.1111/1469-8986.00172.x 15102118

[49] Marques C, Moreno S, Luís Castro S, Besson M. Musicians detect pitch violation in a foreign language better than nonmusicians: behavioral and electrophysiological evidence. J Cogn Neurosci. 2007; 19: 1453–1463. doi: 10.1162/jocn.2007.19.9.1453 17714007

[50] Deguchi C, Boureux M, Sarlo M, Besson M, Grassi M, Schön D, et al. Sentence pitch change detection in the native and unfamiliar language in musicians and non-musicians: Behavioral, electrophysiological and psychoacoustic study. Brain Res. 2012; 1455: 75–89. doi: 10.1016/j.brainres.2012.03.034 22498174

[51] Baills F, Zhang Y, Cheng Y, Bu Y, Prieto P. Listening to Songs and Singing Benefitted Initial Stages of Second Language Pronunciation but Not Recall of Word Meaning. Lang Learn. 2021; 71: 369–413. 10.1111/lang.12442

[52] Cooper A, Wang Y, Ashley R. Thai rate-varied vowel length perception and the impact of musical experience. Lang Speech. 2017; 60: 65–84. doi: 10.1177/0023830916642489 28326992

[53] Alexander JA, Wong PCM, Bradlow AR. Lexical tone perception in musicians and non-musicians. 9th European Conference on Speech Communication and Technology. Lisbon, Portugal; 2005. pp. 397–400. http://www.isca-speech.org/archive/interspeech_2005/i05_0397.html

[54] Burnham DK, Brooker R. Absolute pitch and lexical tones: Tone perception by non-musician, musician, and absolute pitch non-tonal language speakers. Seventh International Conference on Spoken Language Processing. Denver, Colorado, USA; 2002. pp. 257–260. 10.21437/ICSLP.2002-129

[55] Fuller CD, Galvin JJ III, Maat B, Free RH, Başkent D. The musician effect: does it persist under degraded pitch conditions of cochlear implant simulations? Front Neurosci. 2014; 8: 179. doi: 10.3389/fnins.2014.00179 25071428 PMC4075350

[56] Besson M, Chobert J, Marie C. Transfer of training between music and speech: Common processing, attention, and memory. Front Psychol. 2011; 2: 1–12. 10.3389/fpsyg.2011.00094 21738519 PMC3125524

[57] Başkent D, Gaudrain E. Musician advantage for speech-on-speech perception. J Acoust Soc Am. 2016; 139: EL51–EL56. doi: 10.1121/1.4942628 27036287

[58] Nagle C. A reexamination of ultimate attainment in L2 phonology: Length of immersion, motivation, and phonological short-term memory. Cascadilla Proceedings Project. Sommerville, MA, USA; 2013. pp. 148–161.

[59] Shively RL. L2 acquisition of [β], [δ], and [γ] in Spanish: Impact of experience, linguistic environment and learner variables. Southwest Journal of Linguistics. 2008; 27.

[60] Félix-Brasdefer JC. Data collection methods in speech act performance. Speech act performance: Theoretical, empirical and methodological issues. 2010; 26: 69–82. 10.1075/lllt.26.03fel

[61] Open AI. 2021 Jan 5 [cited 12 Nov 2023]. In: DALL-E [Image Generation Model]. Available from https://chat.openai.com/chat

[62] Borràs-Comes J, Sichel-Bazin R, Prieto P. Vocative intonation preferences are sensitive to politeness factors. Lang Speech. 2015; 58: 68–83. doi: 10.1177/0023830914565441 25935938

[63] Alonso MC BMJL. Cantabrian Spanish Intonation. In: Prieo Pilar, Roseano Paolo, editors. Transcription of Intonation of the Spanish Language. Lincom Europa; 2010. pp. 49–85. 10.1515/shll-2011-1109

[64] Alonso MC, Bobo MJL. Dialectos en contacto y prosodia. Análisis contrastivo de la entonación del oriente y occidente de Cantabria. Revista internacional de lingüística iberoamericana. 2011; 39–51. 10.2307/41670571

[65] Estebas-Vilaplana E, Prieto P. La notación prosódica del español: una revisión del Sp-ToBI. Estudios de fonética experimental. 2008; 17: 264–283. http://hdl.handle.net/10230/27258

[66] Face TL. Perception of Castilian Spanish Intonation: Implications for Intonational Phonology. Munich: Lincom Europa; 2011.

[67] Boersma P, Weenink D. Praat: doing phonetics by computer (Version 5.3.82). 2020. Available from: http://www.praat.org/

[68] Yuan C, González-Fuente S, Baills F, Prieto P. Observing pitch gestures favors the learning of spanish intonation by mandarin speakers. Stud Second Lang Acquis. 2019; 41: 5–32. 10.1017/S02722631170003166

[69] Law LNC, Zentner M. Assessing musical abilities objectively: Construction and validation of the Profile of Music Perception Skills. PLoS One. 2012; 7: e52508. doi: 10.1371/journal.pone.0052508 23285071 PMC3532219

[70] Baron-Cohen S, Wheelwright S, Skinner R, Martin J, Clubley E. Tone Language Speakers and Musicians Share Enhanced Perceptual and Cognitive Abilities for Musical Pitch: Evidence for Bidirectionality between the Domains of Language. J Autism Dev Disord. 2001; 31: 5–17. 10.1371/journal.pone.0060676 11439754

[71] Lau WYP, Gau SSF, Chiu YN, Wu YY, Chou WJ, Liu SK, et al. Psychometric properties of the Chinese version of the Autism Spectrum Quotient (AQ). Res Dev Disabil. 2013; 34: 294–305. doi: 10.1016/j.ridd.2012.08.005 22985783

[72] Liu M. Chinese Version of the Autism Spectrum Quotient for Adults: Predictive Validity and Correlation Analysis. Journal of Special Education Research. 2008; 33: 73–92.

[73] Ripley B, Venables B, Bates DM, Hornik K, Gebhardt A, Firth D, et al. Package ‘mass.’ Cran r. 2013; 538: 113–120. https://cran.r-project.org/package=MASS

[74] Lenth R, Singmann H, Love J, Buerkner P, Herve M. Emmeans: Estimated marginal means, aka least-squares means. R package version. 2018; 1: 3. https://cran.r-project.org/package=emmeans

[75] R Core Team. R: A language and environment for statistical computing. Vienna, Austria: R Foundation for Statistical Computing; 2020. https://www.r-project.org/

[76] Trimble JC. Perceiving Intonational Cues in a Foreign Language: Perception of Sentence Type in Two Dialects of Spanish. In: Howe C, Blackwell SE, Quesada ML, editors. 15th Hispanic Linguistics Symposioum. Athens, USA; 2013. pp. 78–92.

[77] Camacho J. Introducción a la sintaxis del español. Cambridge University Press; 2018.

[78] Ituarte AL. Clause type vs speech act: Knowledge confirmation questions in Basque. J Linguist. 2022; 1–27. 10.1017/S0022226722000469

[79] Bacskai-Atkari J. Clause Types (and Clausal Complementation) in Germanic. Oxford Research Encyclopedia of Linguistics. 2022. 10.1093/acrefore/9780199384655.013.978

[80] Gussenhoven C, Chen A. Universal and language-specific effects in the perception of question intonation. 6th International Conference on Spoken Language Processing. Beijing, China; 2000. pp. 91–94.

[81] Chen A, Rietveld T, Gussenhoven C. Language-specific effects of pitch range on the perception of universal intonational meaning. 7th European Conference on Speech Communication and Technology. Aalborg, Denmark; 2001. pp. 1403–1406.

[82] Kraut R, Wulff S. Foreign-accented speech perception ratings: A multifactorial case study. J Multiling Multicult Dev. 2013; 34: 249–263. 10.1080/01434632.2013.767340

[83] Smiljanić R, Bradlow AR. Bidirectional clear speech perception benefit for native and high-proficiency non-native talkers and listeners: Intelligibility and accentedness. J Acoust Soc Am. 2011; 130: 4020–4031. doi: 10.1121/1.3652882 22225056 PMC3253601

[84] Bidelman GM, Gandour JT, Krishnan A. Cross-domain effects of music and language experience on the representation of pitch in the human auditory brainstem. J Cogn Neurosci. 2011; 23: 425–434. doi: 10.1162/jocn.2009.21362 19925180

