# Supplementary material for: Unraveling the contributions of prosodic patterns and individual traits on cross-linguistic perception of Spanish sentence modality
Source: PLoS One. 2024 Feb 29;19(2):e0298708. doi: 10.1371/journal.pone.0298708 (PMC10903904; doi:10.1371/journal.pone.0298708)
Supplement: S1 Appendix — (PPTX) [file pone.0298708.s001.pptx]

## Slide 1
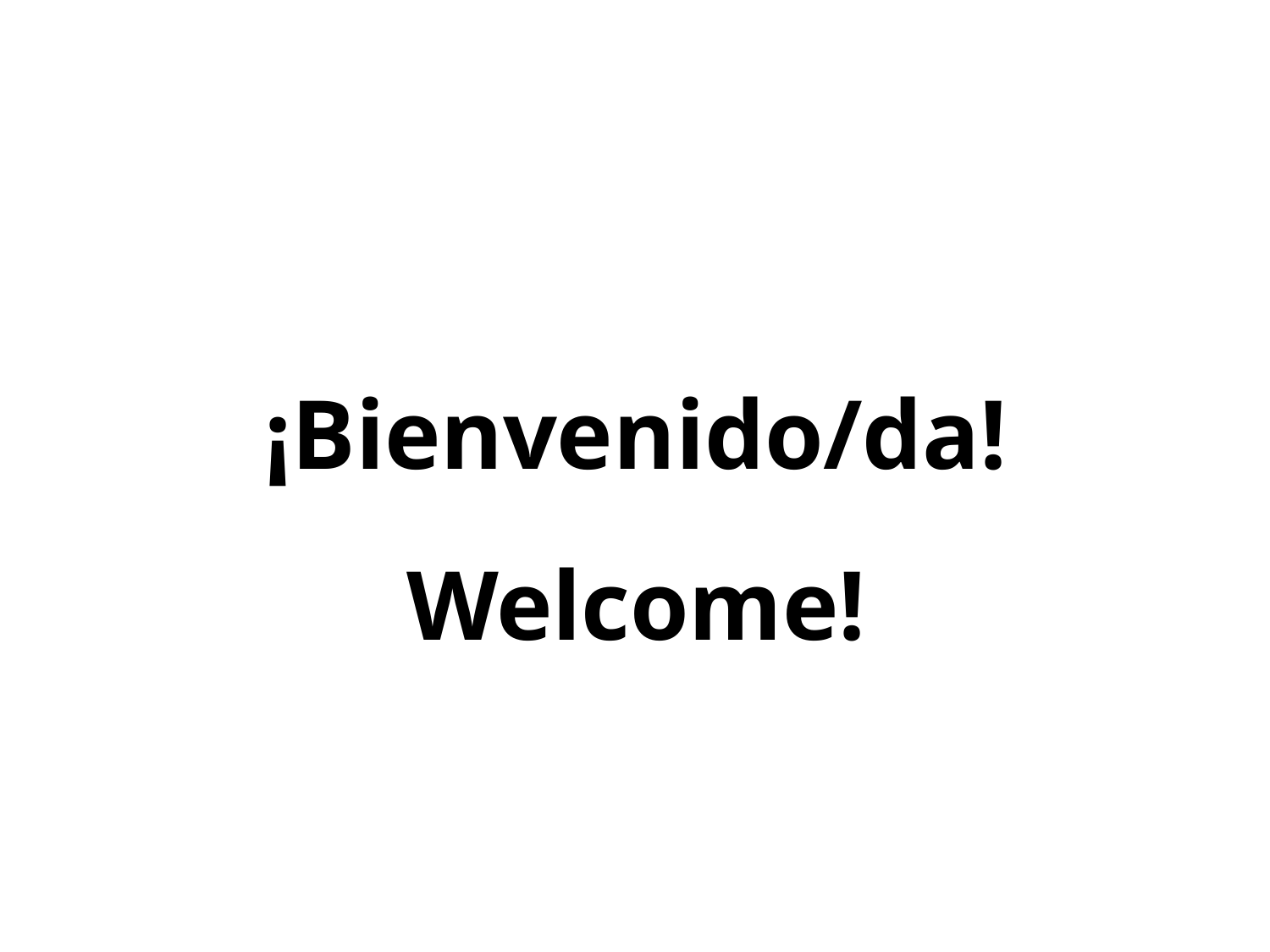

# ¡Bienvenido/da!
Welcome!

## Slide 2
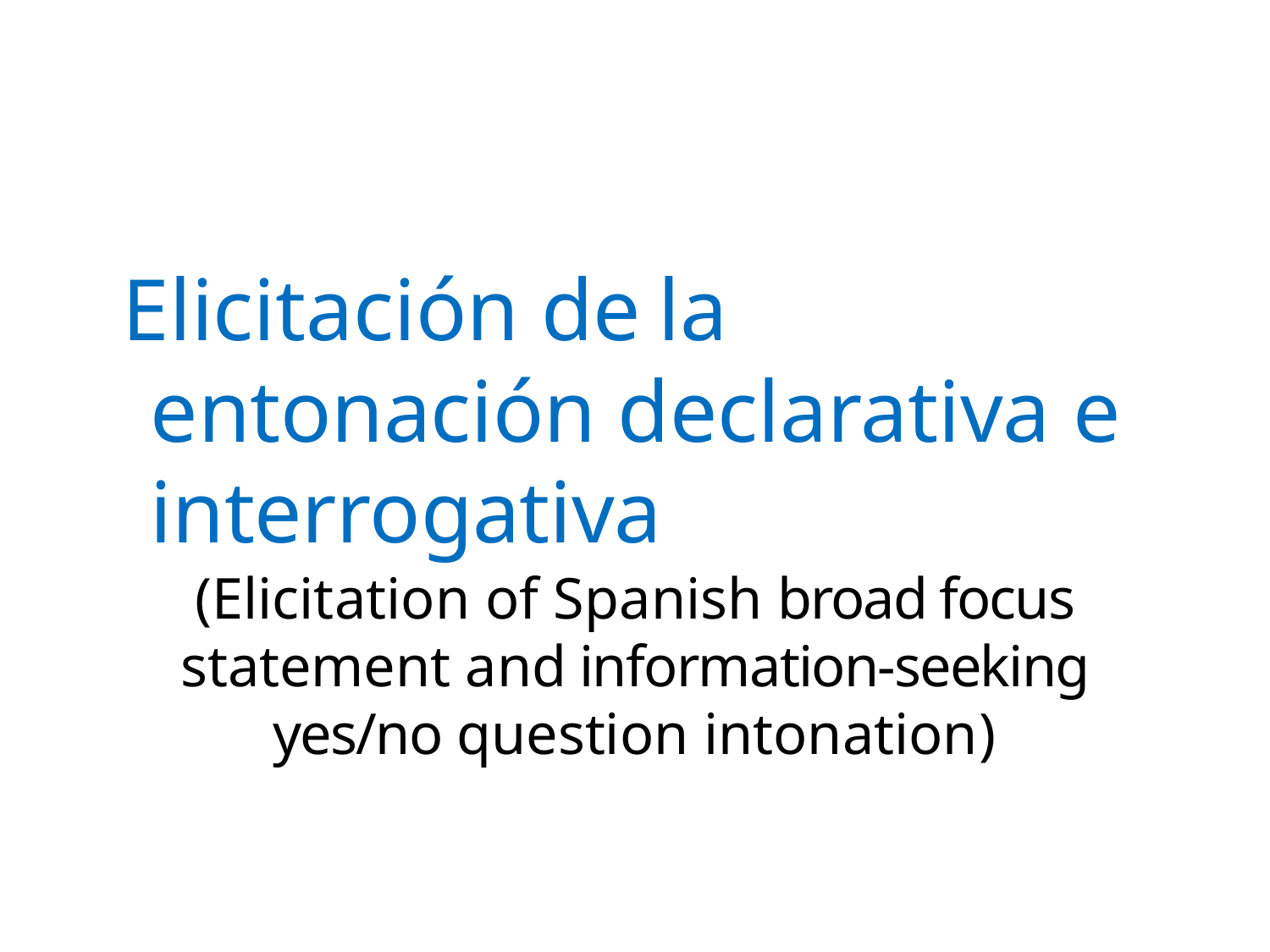

# Elicitación de	la entonación declarativa e interrogativa
(Elicitation of Spanish broad focus statement and information-seeking yes/no question intonation)

## Slide 3
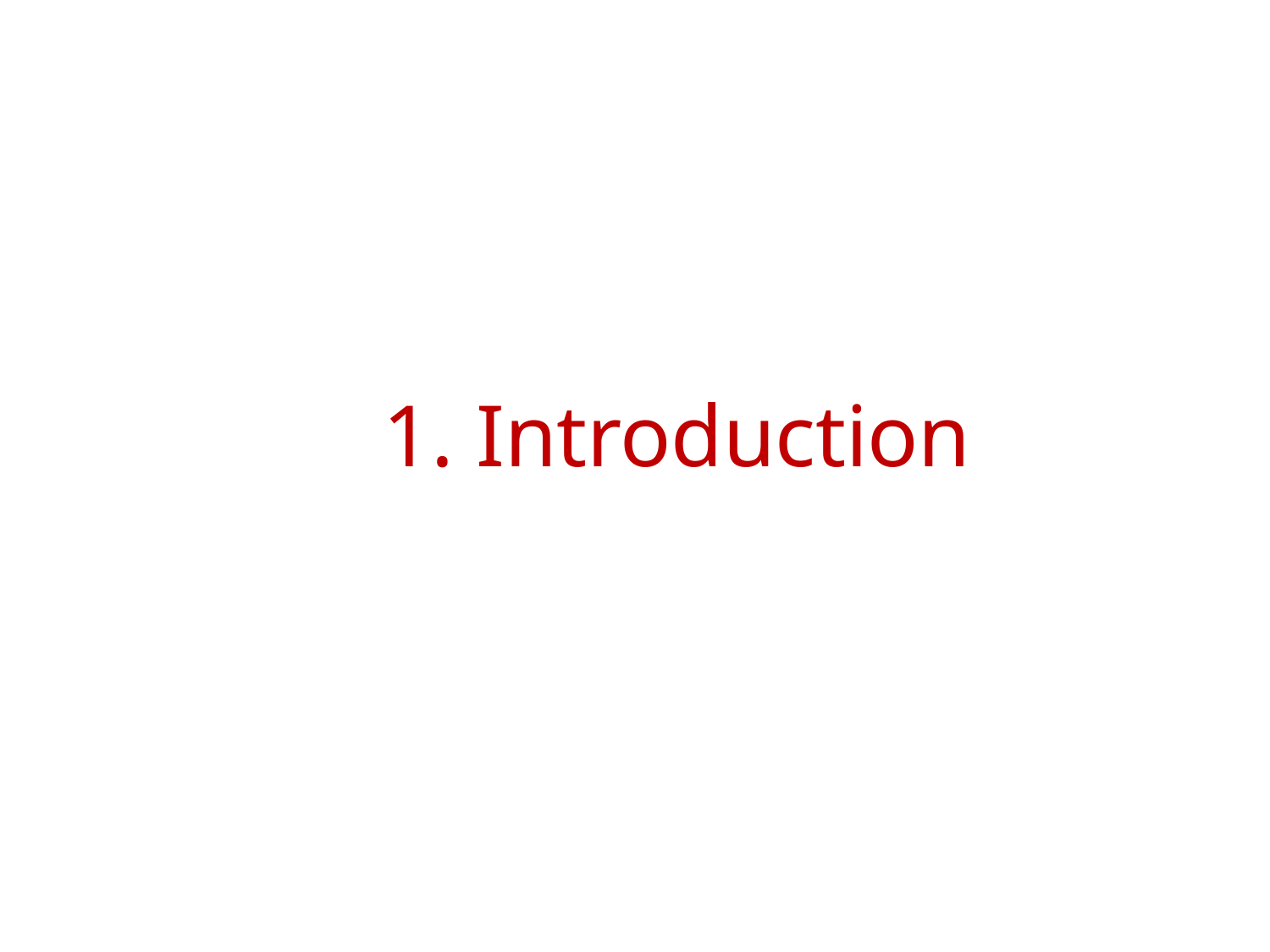

# 1. Introduction

## Slide 4
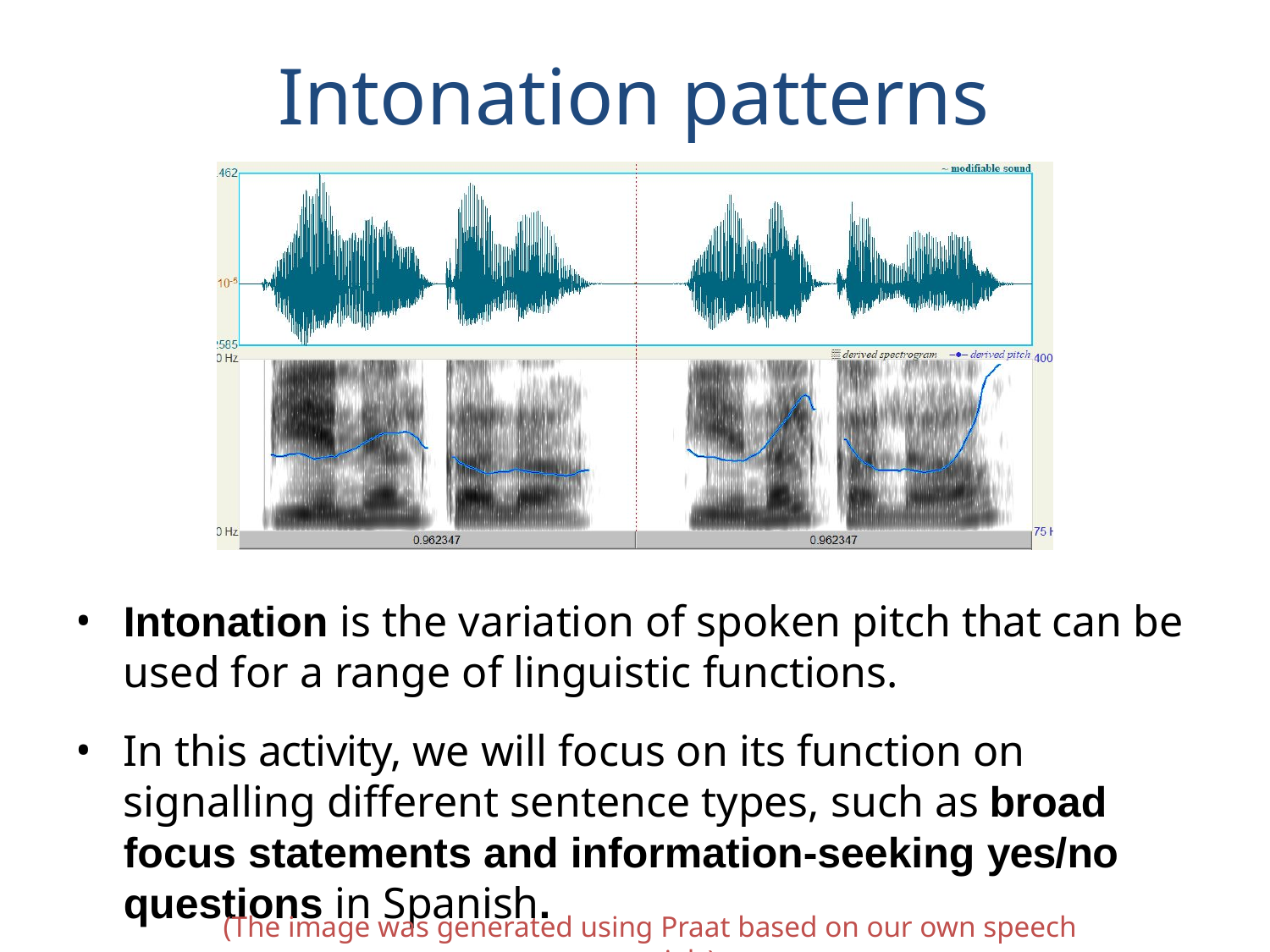

# Intonation patterns
Intonation is the variation of spoken pitch that can be used for a range of linguistic functions.
In this activity, we will focus on its function on signalling different sentence types, such as broad focus statements and information-seeking yes/no questions in Spanish.
(The image was generated using Praat based on our own speech materials)

## Slide 5
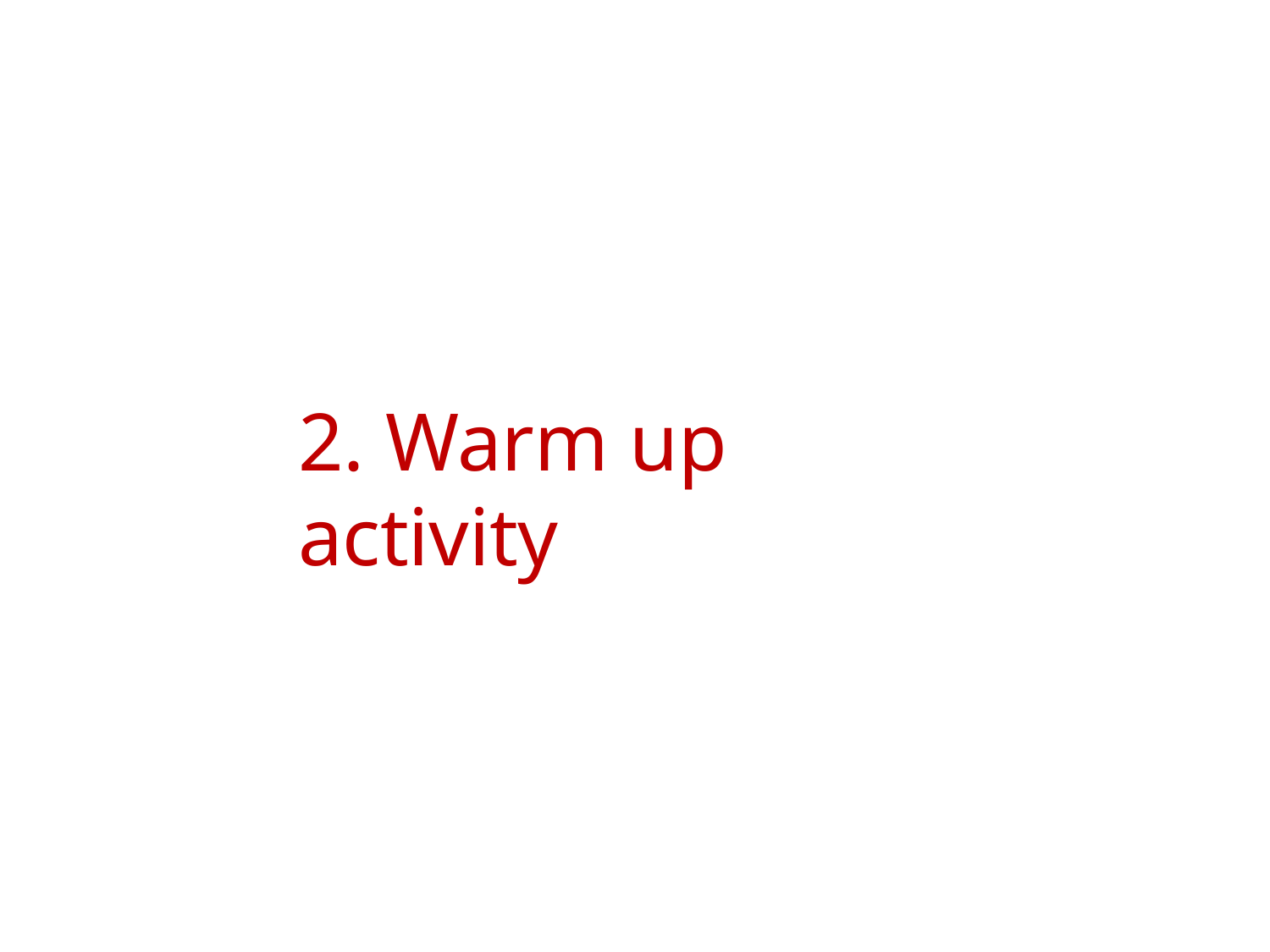

# 2. Warm up activity

## Slide 6
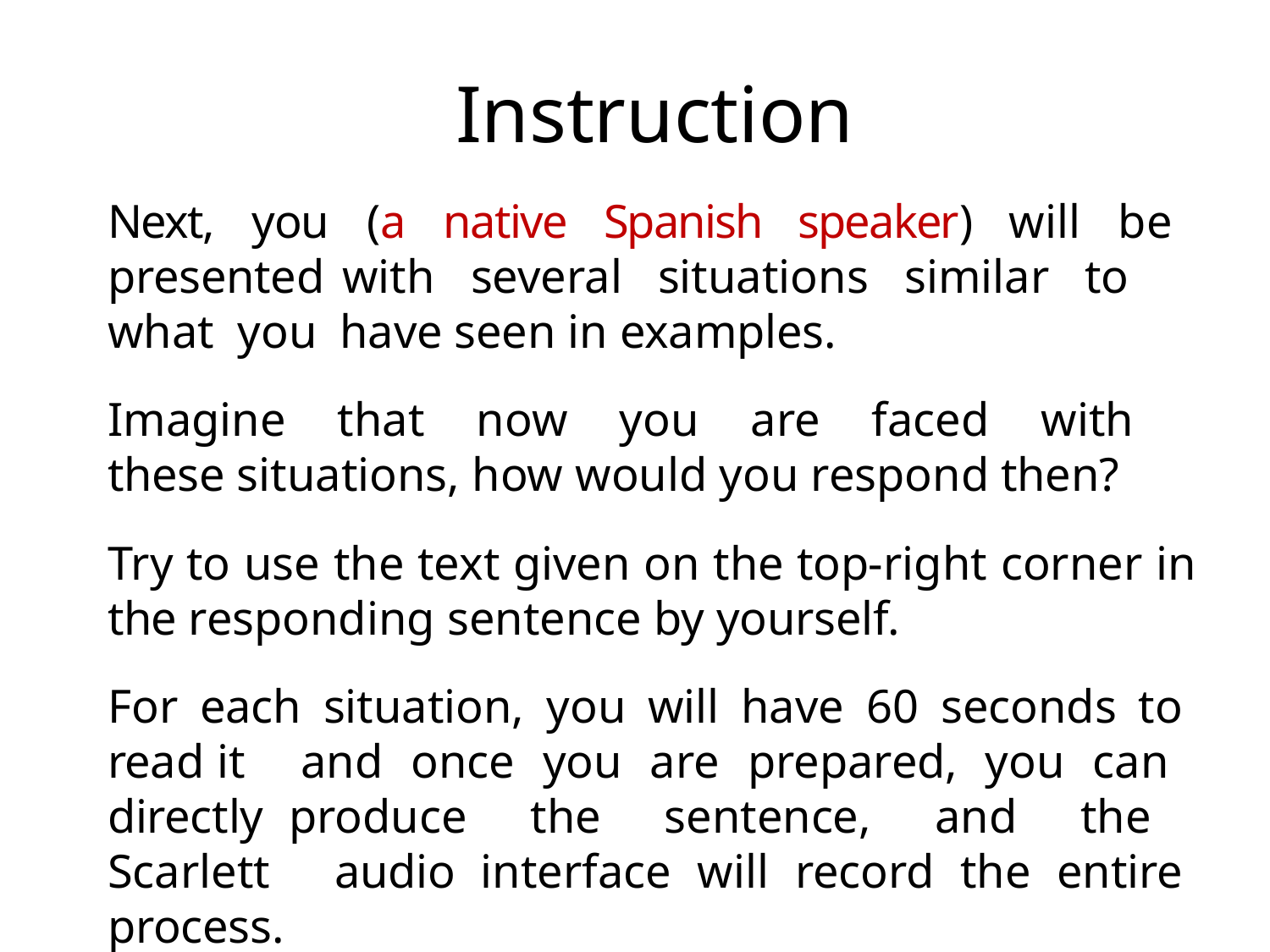

# Instruction
Next, you (a native Spanish speaker) will be presented with several situations similar to what you have seen in examples.
Imagine that now you are faced with these situations, how would you respond then?
Try to use the text given on the top-right corner in the responding sentence by yourself.
For each situation, you will have 60 seconds to read it and once you are prepared, you can directly produce the sentence, and the Scarlett audio interface will record the entire process.

## Slide 7
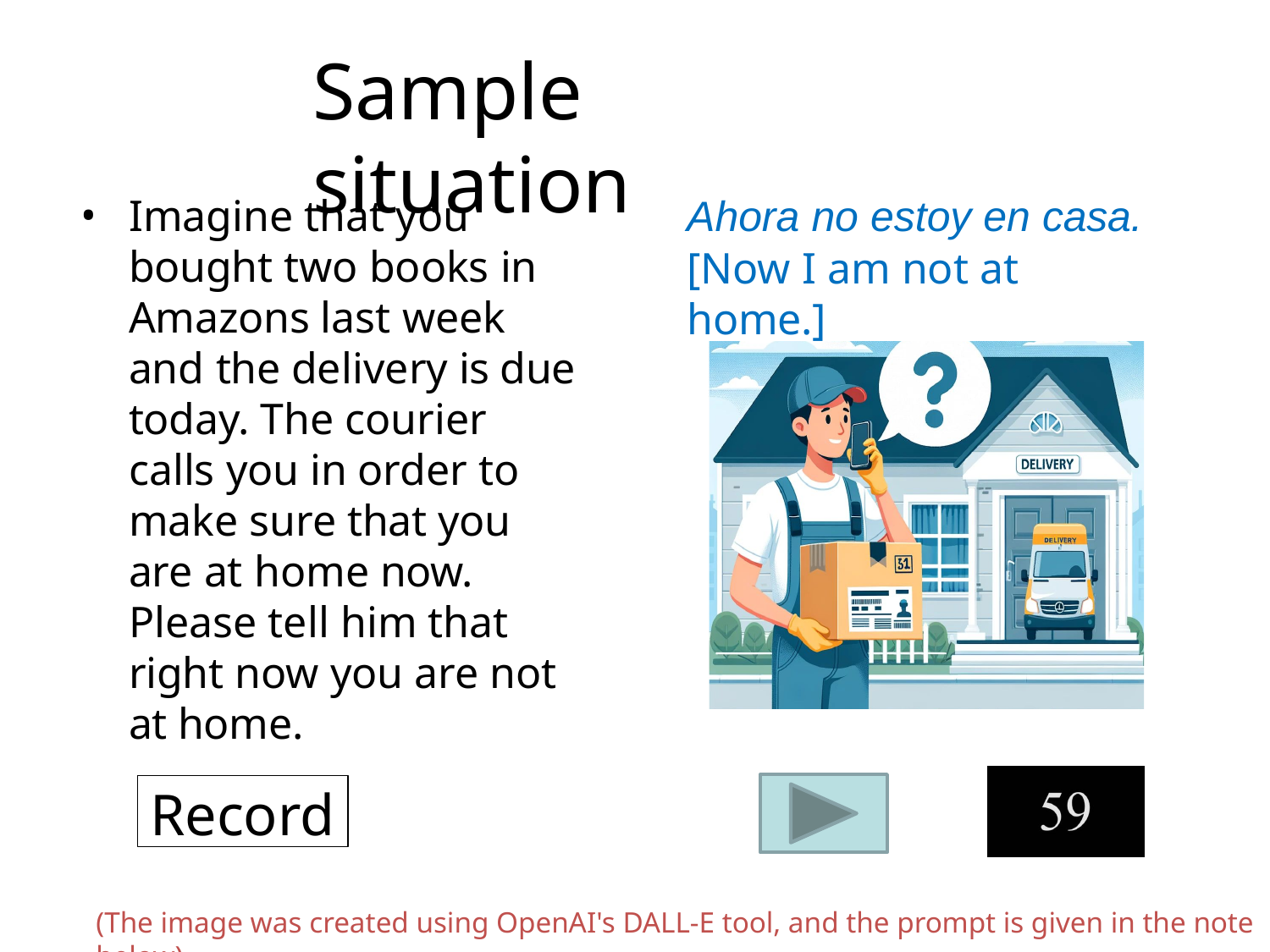

# Sample situation
Imagine that you bought two books in Amazons last week and the delivery is due today. The courier calls you in order to make sure that you are at home now. Please tell him that right now you are not at home.
Ahora no estoy en casa.
[Now I am not at home.]
Record
(The image was created using OpenAI's DALL-E tool, and the prompt is given in the note below)

## Slide 8
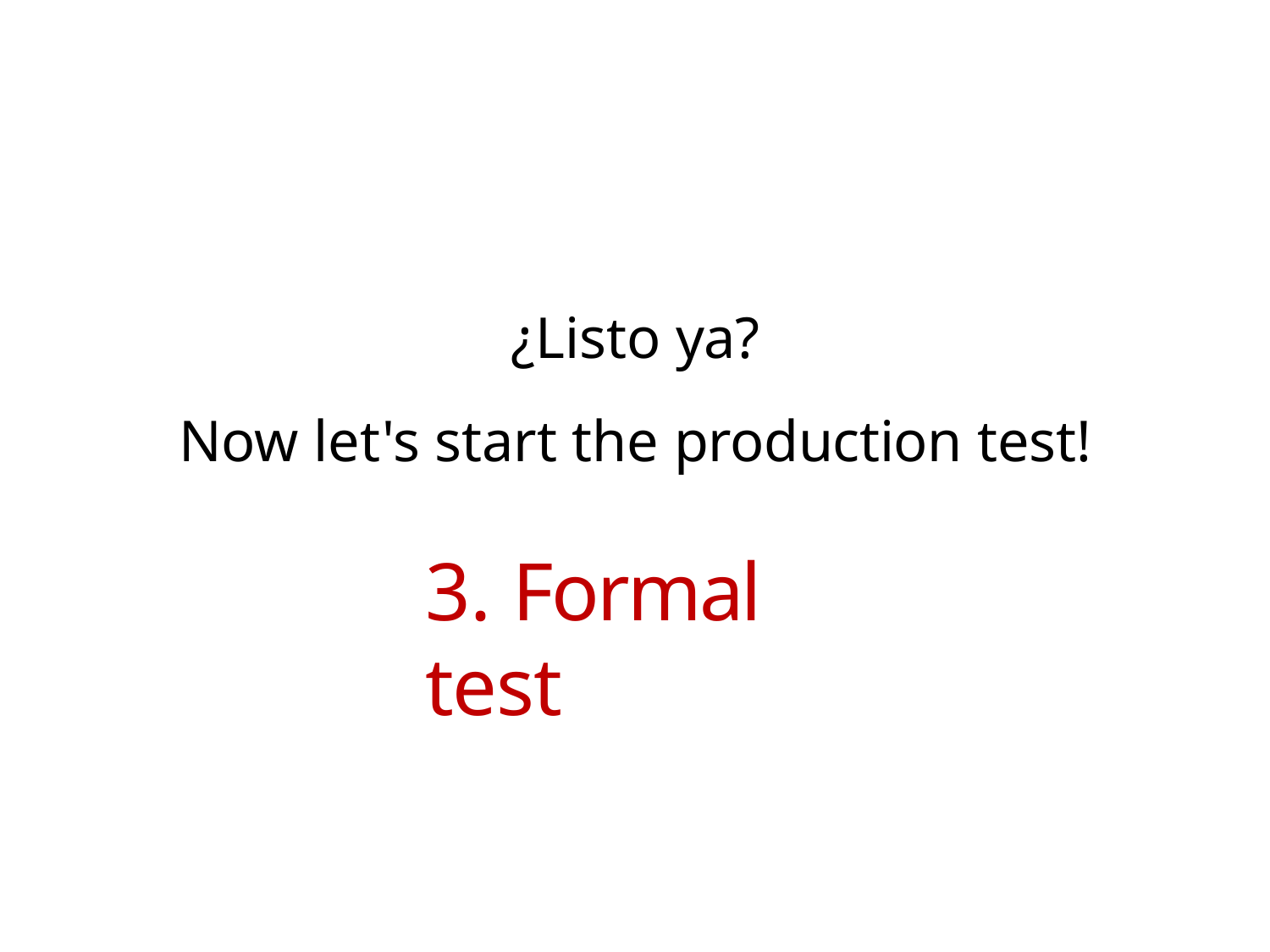

# ¿Listo ya?
Now let's start the production test!
3. Formal test

## Slide 9
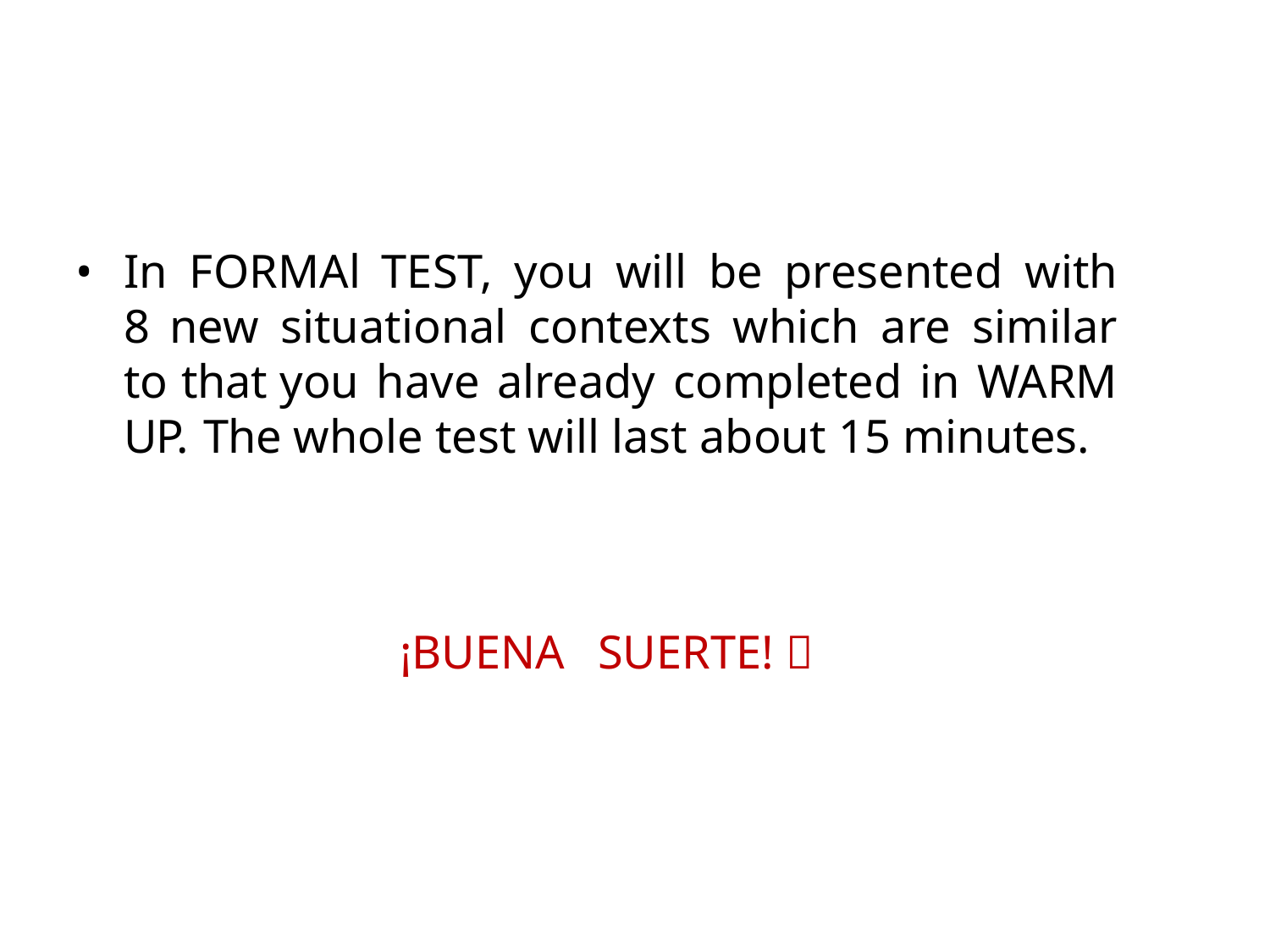

In FORMAl TEST, you will be presented with 8 new situational contexts which are similar to that you have already completed in WARM UP. The whole test will last about 15 minutes.
¡BUENA	SUERTE! 💪

## Slide 10
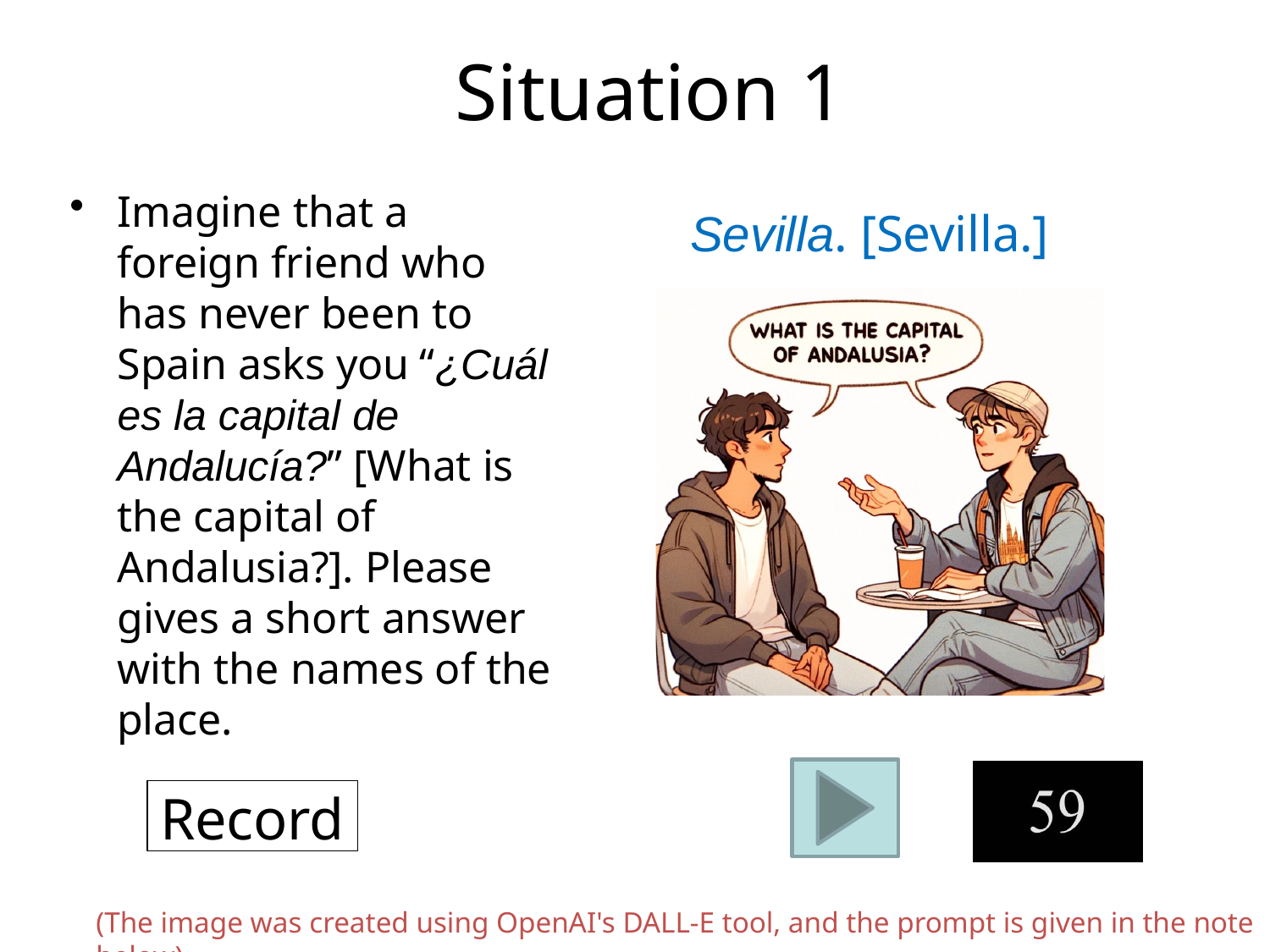

# Situation 1
Imagine that a foreign friend who has never been to Spain asks you “¿Cuál es la capital de Andalucía?” [What is the capital of Andalusia?]. Please gives a short answer with the names of the place.
Sevilla. [Sevilla.]
Record
(The image was created using OpenAI's DALL-E tool, and the prompt is given in the note below)

## Slide 11
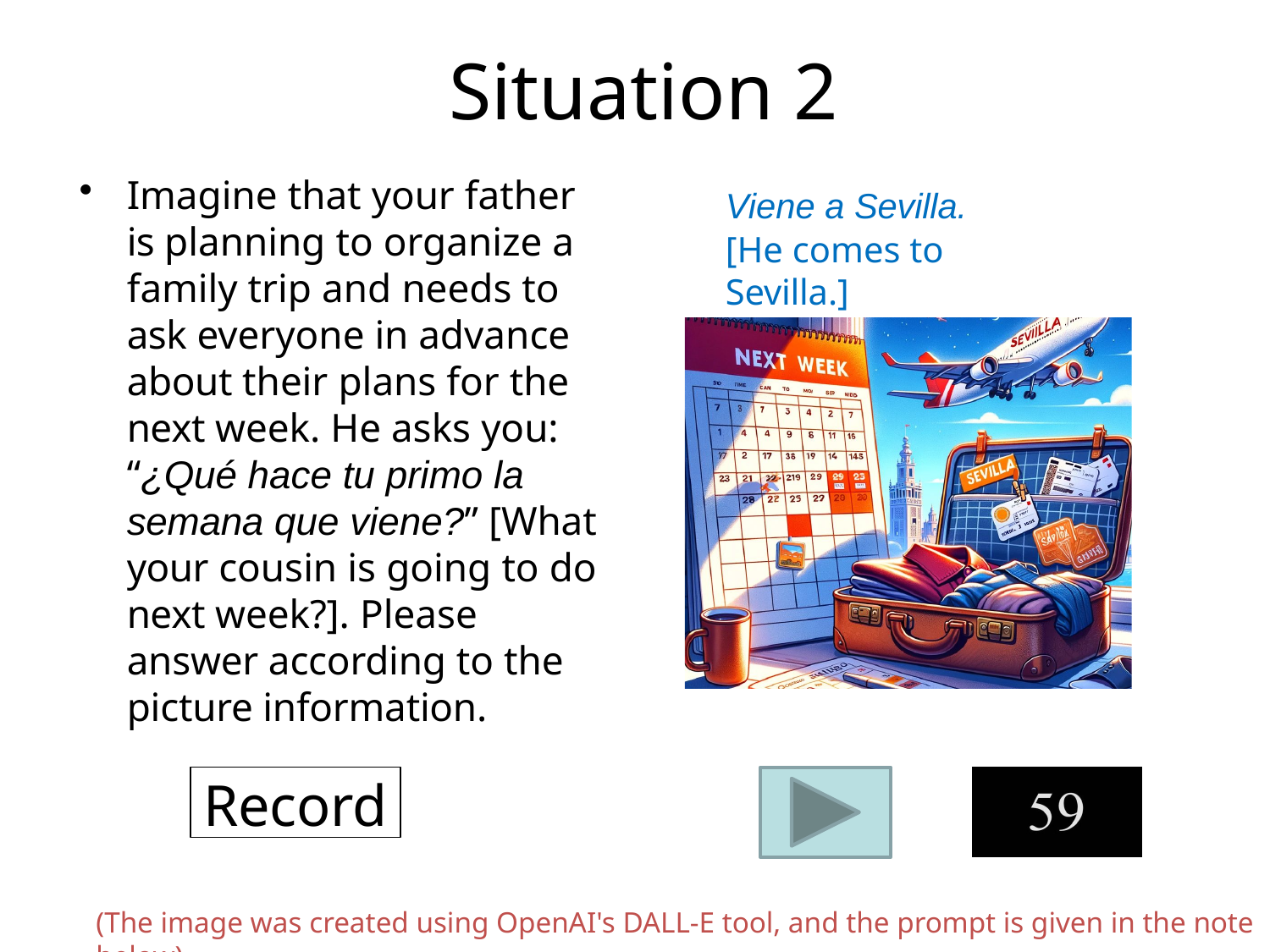

# Situation 2
Imagine that your father is planning to organize a family trip and needs to ask everyone in advance about their plans for the next week. He asks you: “¿Qué hace tu primo la semana que viene?” [What your cousin is going to do next week?]. Please answer according to the picture information.
Viene a Sevilla.
[He comes to Sevilla.]
Record
(The image was created using OpenAI's DALL-E tool, and the prompt is given in the note below)

## Slide 12
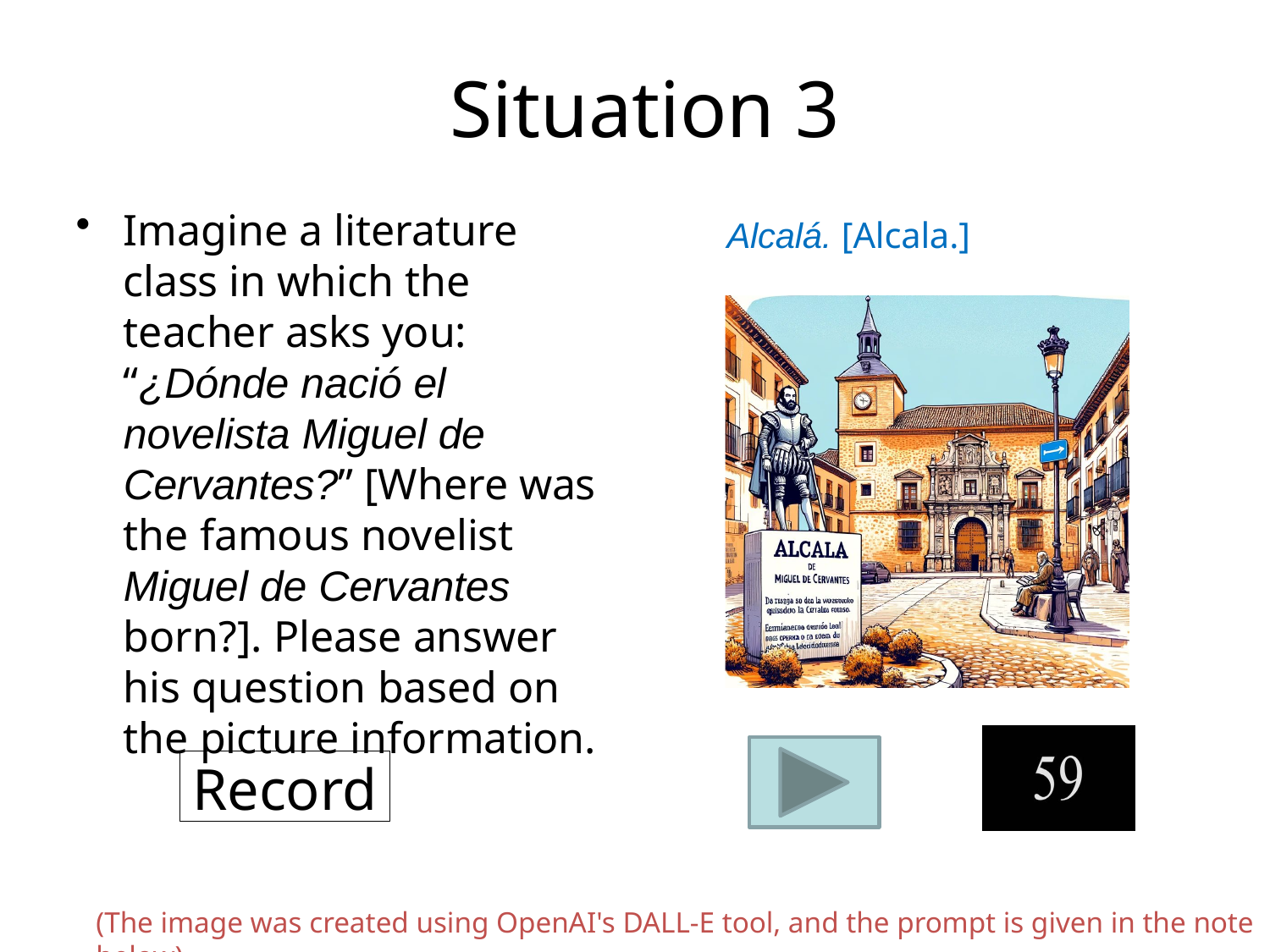

# Situation 3
Imagine a literature class in which the teacher asks you: “¿Dónde nació el novelista Miguel de Cervantes?” [Where was the famous novelist Miguel de Cervantes born?]. Please answer his question based on the picture information.
Alcalá. [Alcala.]
Record
(The image was created using OpenAI's DALL-E tool, and the prompt is given in the note below)

## Slide 13
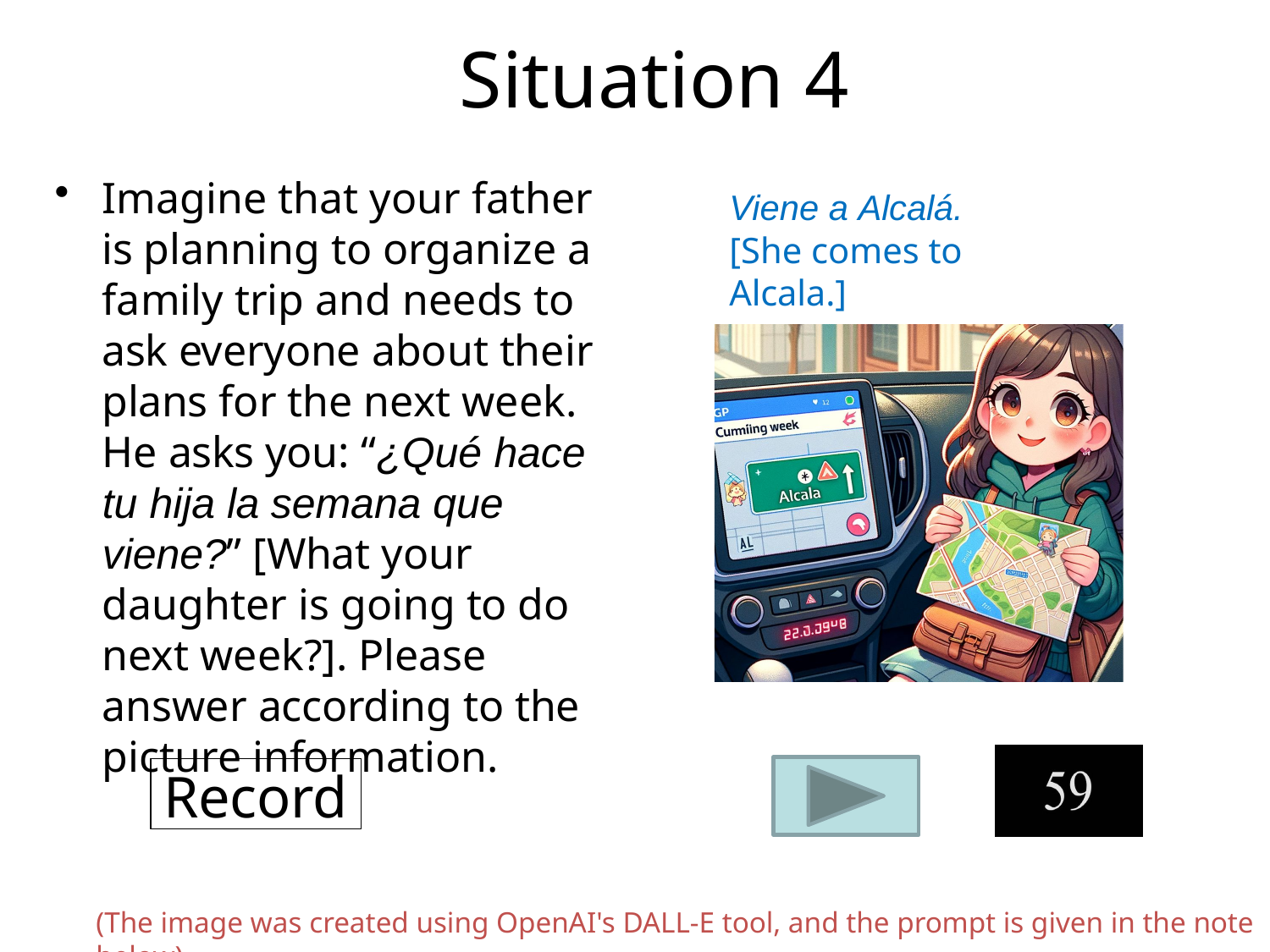

# Situation 4
Imagine that your father is planning to organize a family trip and needs to ask everyone about their plans for the next week. He asks you: “¿Qué hace tu hija la semana que viene?” [What your daughter is going to do next week?]. Please answer according to the picture information.
Viene a Alcalá.
[She comes to Alcala.]
Record
(The image was created using OpenAI's DALL-E tool, and the prompt is given in the note below)

## Slide 14
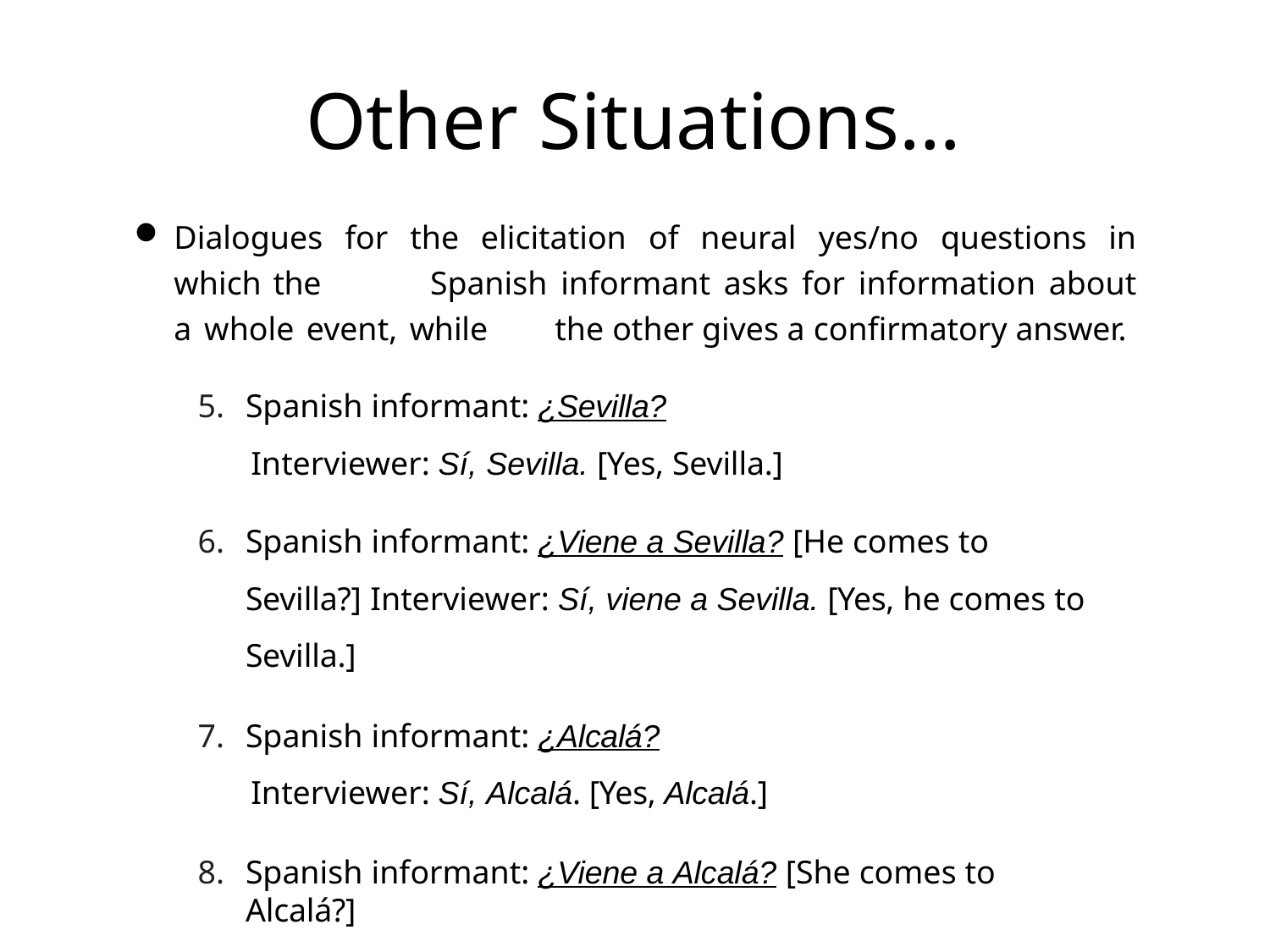

# Other Situations…
Dialogues for the elicitation of neural yes/no questions in which the 	Spanish informant asks for information about a whole event, while 	the other gives a confirmatory answer.
Spanish informant: ¿Sevilla?
Interviewer: Sí, Sevilla. [Yes, Sevilla.]
Spanish informant: ¿Viene a Sevilla? [He comes to Sevilla?] 	Interviewer: Sí, viene a Sevilla. [Yes, he comes to Sevilla.]
Spanish informant: ¿Alcalá?
Interviewer: Sí, Alcalá. [Yes, Alcalá.]
Spanish informant: ¿Viene a Alcalá? [She comes to Alcalá?]
Interviewer: Sí, viene a Alcalá. [Yes, she comes to Alcalá.]

## Slide 15
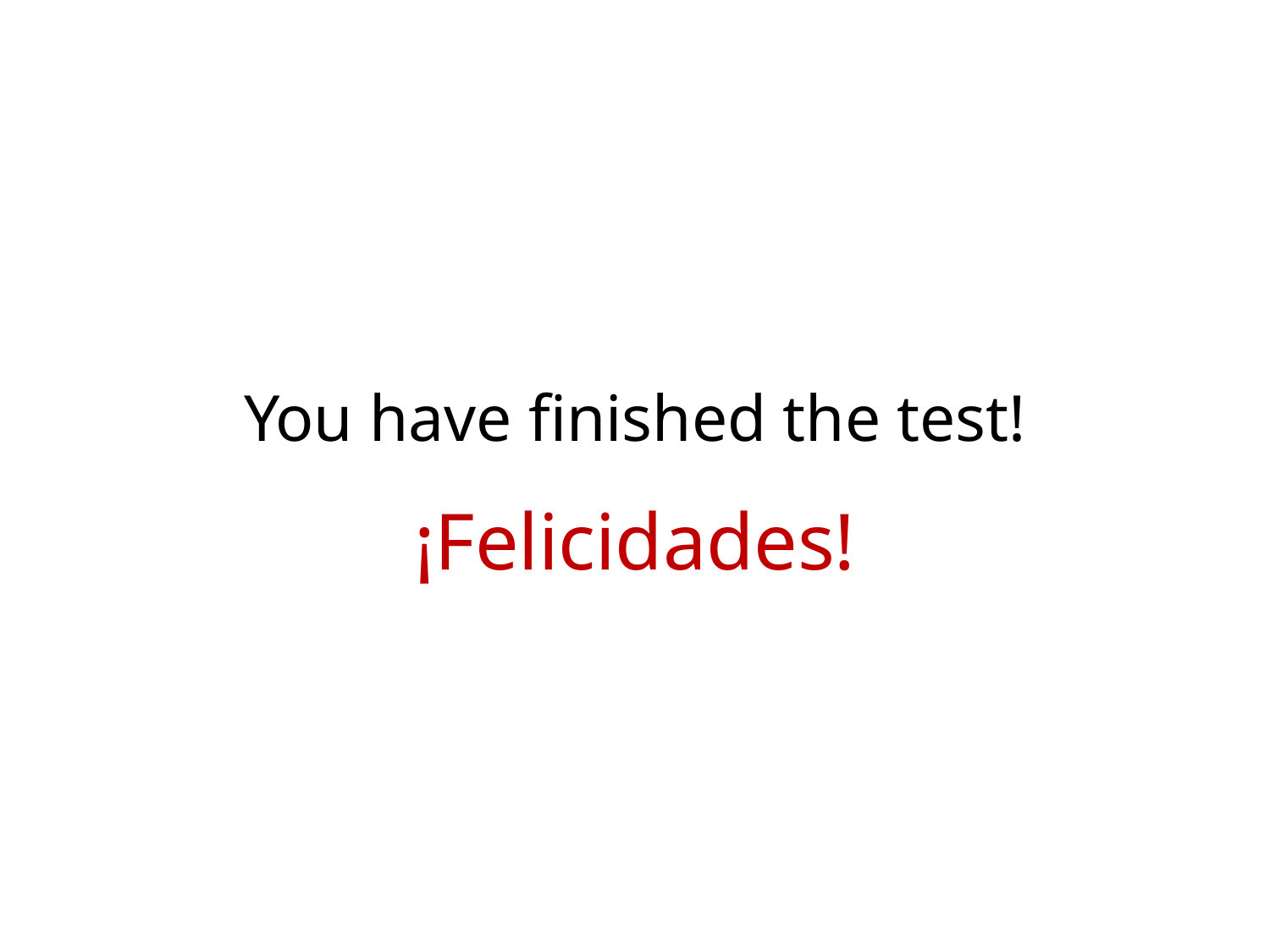

# You have finished the test!
¡Felicidades!

## Slide 16
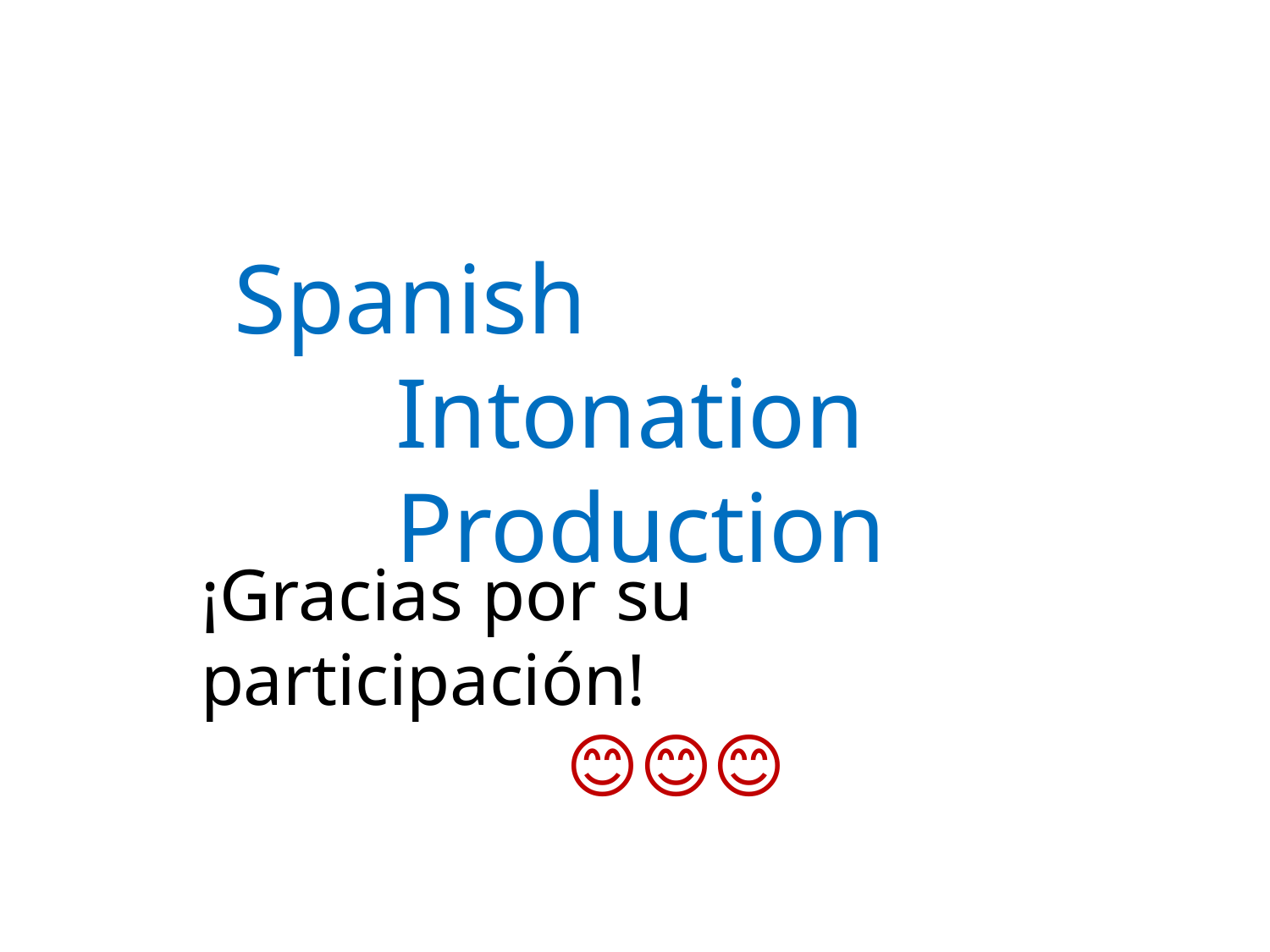

# Spanish Intonation Production
¡Gracias por su participación!
😊😊😊
