# Supplementary material for: Unraveling the contributions of prosodic patterns and individual traits on cross-linguistic perception of Spanish sentence modality
Source: PLoS One. 2024 Feb 29;19(2):e0298708. doi: 10.1371/journal.pone.0298708 (PMC10903904; doi:10.1371/journal.pone.0298708)
Supplement: S2 Appendix — (ZIP) [file pone.0298708.s002.zip › S2 Appendix. Audio files/Stimulus list with identifier.pdf]

S2 Appendix. Audio files of the stimuli created for the study.

List of the stimuli with identifier: The first digit [S/Q] of the identifier indicates the sentence modality [statement/question); the second digit [1/2] indicates the number of stressed words in the utterance. The fourth digit [O/P] indicates the stress pattern of the last word [oxytone/paroxytone], and the last digit [1, 2, 3, 4] indicates the number of gates of the stimulus. **Please find the corresponding audio clip for each stimulus in the sub-folder named "Audio clips."**

Audio name	Stimulus
S1-O1	*Alca…*
S1-O2	*Alcalá.*
S1-P1	*Sevi…*
S1-P2	*Sevilla.*
S2-O1	*Vie…*
S2-O2	*Viene a al…*
S2-O3	*Viene a alca…*
S2-O4	*Viene a Alcalá.*
S2-P1	*Vie…*
S2-P2	*Viene a…*
S2-P3	*Viene a Sevi…*
S2-P4	*Viene a Sevilla.*
Q1-O1	*¿Alca…?*
Q1-O2	*¿Alcalá?*
Q1-P1	*¿Sevi…?*
Q1-P2	*¿Sevilla?*
Q2-O1	*¿Vie…?*
Q2-O2	*¿Viene a al…?*
Q2-O3	*¿Viene a alca…?*
Q2-O4	*¿Viene a Alcalá?*
Q2-P1	*¿Vie…?*
Q2-P2	*¿Viene a…?*
Q2-P3	*¿Viene a Sevi…?*
Q2-P4	*¿Viene a Sevilla?*
