# Supplementary material for: Unraveling the contributions of prosodic patterns and individual traits on cross-linguistic perception of Spanish sentence modality
Source: PLoS One. 2024 Feb 29;19(2):e0298708. doi: 10.1371/journal.pone.0298708 (PMC10903904; doi:10.1371/journal.pone.0298708)
Supplement: S3 Appendix — (PDF) [file pone.0298708.s003.pdf]

S3 Appendix. Autism Spectrum Quotient (AQ) items in three languages.

Items on the Communication Subscale of the Autism Spectrum Quotient (AQ) (Baron-Cohen et al., 2001a, 2001b). Higher levels of autistic traits related to communication (and thus lower pragmatic skill) are assigned when participants respond with "slightly agree" and "strongly agree" to the items 1–6, or with "slightly disagree" and "strongly disagree" to the items 7–10. Below are the translations of the ten AQ items, which were originally in English, into Spanish and Chinese.

Table 1. AQ items of the communication subscale in English.

		Slightly agree	Strongly agree	Slightly disagree	Strongly disagree
1	Other people frequently tell me that what I've said is impolite, even though I think it is polite.	○	○	○	○
2	I enjoy social chit-chat.	○	○	○	○
3	When I talk, it isn't always easy for others to get a word in edgeways.	○	○	○	○
4	I frequently find that I don't know how to keep a conversation going.	○	○	○	○
5	I find it easy to "read between the lines" when someone is talking to me.	○	○	○	○
6	I know how to tell if someone listening to me is getting bored.	○	○	○	○
7	When I talk on the phone, I'm not sure when it's my turn to speak.	○	○	○	○
8	I am often the last to understand the point of a joke.	○	○	○	○
9	I am good at social chit-chat.	○	○	○	○

10	People often tell me that I keep going on and on about the same thing.	○	○	○	○

Table 2. Spanish translation of the AQ items in the communication subscale.

		Acuerdo total	Acuerdo parcial	Desacuerdo parcial	Desacuerdo total
1	A menudo otras personas me dicen que lo que he dicho es maleducado, a pesar de que yo en realidad no creo que sea así.	○	○	○	○
2	Me gusta la cháchala social.	○	○	○	○
3	Cuando yo hablo no siempre es fácil para los demás meter la pata.	○	○	○	○
4	A menudo me ocurre que no sé cómo mantener una conversación. con otra persona.	○	○	○	○
5	Me resulta fácil “leer entre líneas” o captar el doble sentido, cuando alguien me está hablando.	○	○	○	○
6	Soy capaz de darme cuenta si una persona que me está escuchando se aburre.	○	○	○	○
7	Cuando hablo por teléfono no estoy seguro de cuando es mi turno para hablar.	○	○	○	○
8	A menudo soy el último en entender la gracia de un chiste.	○	○	○	○
9	Soy bueno en las charlas	○	○	○	○

	sociales.				
10	La gente a menudo me dice que vuelvo una y otra vez sobre el mismo tema.	○	○	○	○

Table 3. Chinese translation of the AQ items in the communication subscale.

		完全同意	稍微同意	稍微不同意	完全不同意
1	虽然我自认为很有礼貌，但还是经常被其他人告知我说了不礼貌的话。	○	○	○	○
2	我喜欢社交闲谈。	○	○	○	○
3	当我说话时，别人常常不是很容易能插得上话。	○	○	○	○
4	我时常发现我不知如何使对话持续下去。	○	○	○	○
5	与人谈话时，我能很轻易地察觉对方的言外之意。	○	○	○	○
6	我知道如何辨别对方是否已经厌倦听我说话。	○	○	○	○
7	当我讲电话时，我不太确定什么时候该我接话	○	○	○	○
8	我常常是最后一个捕捉到笑话笑点的人。	○	○	○	○
9	我擅长社交闲谈。	○	○	○	○
10	我常被告知总是重复地说同样的事。	○	○	○	○
